# Supplementary material for: Aggregation induced emission dynamic chiral europium(III) complexes with excellent circularly polarized luminescence and smart sensors
Source: Nat Commun. 2024 Apr 4;15:2896. doi: 10.1038/s41467-024-47246-z (PMC10994944; doi:10.1038/s41467-024-47246-z)
Supplement: Supplementary file 1 — Supplementary Information [file 41467_2024_47246_MOESM1_ESM.pdf]

Supplementary Information

Aggregation Induced Emission Dynamic Chiral Europium(III) Complexes with Excellent Circularly Polarized Luminescence and Smart Sensors

Yun-Lan Li[1], Hai-Ling Wang[1], Zhong-Hong Zhu[1]*, Yu-Feng Wang[1], Fu-Pei Liang[1]*, and Hua-Hong Zou[1]*

[1]School of Chemistry and Pharmaceutical Sciences, State Key Laboratory for Chemistry and Molecular Engineering of Medicinal Resources, Guangxi Normal University, Guilin 541004, P. R. China
*E-mail (Corresponding author): 18317725515@163.com (Z.-H. Zhu), liangfupei@glut.edu.cn (F.-P. Liang), gxnuchem@foxmail.com (H.-H. Zou).

Table of Supplementary Contents:

**Supplementary Tables**	
**Supplementary Table 1**	Crystallographic data of ***R*/*S*-Eu-R-1** (R = Et/Me), ***R*/*S*-Eu-Et-2**, **Eu-Et-3**, ***R*/*S*-Gd-R-1** (R = Et/Me), ***R*-Gd-Et-2**, ***R*-Tb-Et-1**, ***R*/*S*-Tb-Me-1**, and ***R*-Tb-Et-2**.
**Supplementary Table 2**	*SHAPE* analysis of the Eu^{III} in the ***R*/*S*-Eu-R-1** (R = Et/Me).
**Supplementary Table 3**	*SHAPE* analysis of the Eu^{III} in the ***R*/*S*-Eu-Et-2**.
**Supplementary Table 4**	*SHAPE* analysis of the Eu^{III} in the **Eu-Et-3**.
**Supplementary Table 5**	The fitting comparison of experimental and theoretical values of molecular ion peaks of *R*/*S*-L^1 and *R*/*S*-L^2.
**Supplementary Table 6**	Tabulated photophysical data for ***R*/*S*-Eu-R-1** (R = Et/Me) and ***R*/*S*-Eu-Et-2**.
**Supplementary Table 7**	B_{CPL} value of chiral Eu^{III} complexes.
**Supplementary Figures**	
**Supplementary Figure 1**	Probabilistic ellipsoids and ORTEP-style crystal structure of ***R*/*S*-Eu-R-1** (R = Et/Me) and ***R*/*S*-Eu-Et-2** (A), Ligand coordinated modes and coordinated polyhedron around the metal ions of ***R*/*S*-Eu-R-1** (R = Et/Me) and ***R*/*S*-Eu-Et-2** (B–E).
**Supplementary Figure 2**	Probabilistic ellipsoids and ORTEP-style crystal structure of **Eu-Et-3** (A), Molecular stacking diagram of **Eu-Et-3** (B), Hydrogen bonding of **Eu-Et-3** (C), Ligand coordinated modes (D), Coordinated polyhedron around the metal ions of **Eu-Et-3** (E), Topological structure of **Eu-Et-3** (F).
**Supplementary Figure 3**	IR spectra of ***R*/*S*-Eu-R-1** (R = Et/Me) and ***R*/*S*-Eu-Et-2**.
**Supplementary Figure 4**	Thermogravimetric curves of ***R*/*S*-Eu-R-1** (R = Et/Me) (A–D) and ***R*/*S*-Eu-Et-2** (E and F).
**Supplementary Figure 5**	SEM images of ***R*/*S*-Eu-R-1** (R = Et/Me) and ***R*/*S*-Eu-Et-2**.
**Supplementary Figure 6**	Powder X-ray diffraction patterns (PXRD) of ***R*/*S*-Eu-R-1** (R = Et/Me) (A) and ***R*/*S*-Eu-Et-2** (B).
**Supplementary Figure 7**	QYs of ***R*/*S*-Eu-Et-1** dispersed in glycerin/DMSO (A and B) or CH_3CN/DMF (C and D) mixtures with different f_w.
**Supplementary Figure 8**	QYs of ***R*/*S*-Eu-Et-2** dispersed in glycerin/DMSO (A and B) or CH_3CN/DMF (C and D) mixtures with different f_w.
**Supplementary Figure 9**	QYs of ***R*/*S*-Eu-Me-1** dispersed in glycerin/DMSO (A and B) or CH_3CN/DMF (C and D) mixtures with different f_w.
**Supplementary Figure 10**	Probabilistic ellipsoids and ORTEP-style crystal structure of ***R*/*S*-Gd-R-1** (R = Et/Me) and ***R*-Gd-Et-2** (A) and ***R*-Tb-Et-1/2** and ***R*/*S*-Tb-Me-1** (B).
**Supplementary Figure 11**	Luminescence spectra of ***R*/*S*-Gd-Et-1** and ***R*/*S*-Gd-Et-2** in glycerin/DMSO mixtures with different f_w (A, C, E, and G); Luminescence intensity of ***R*/*S*-Gd-Et-1** and ***R*/*S*-Gd-Et-2** at 443 nm as a function of f_w (the inset shows that the absorption spectra of ***R*/*S*-Gd-Et-1** and ***R*/*S*-Gd-Et-2** in glycerin/DMSO mixed solutions with different contents of f_w) (B, D, F, and H).
**Supplementary Figure 12**	Luminescence spectra of ***R*/*S*-Tb-Et-1** and ***R*/*S*-Tb-Et-2** in glycerin/DMSO mixtures with different f_w (A, C, E, and G); Luminescence intensity of ***R*/*S*-Tb-Et-1** and ***R*/*S*-Tb-Et-2** at 443 nm as a function of f_w (the inset shows that the absorption spectra of ***R*/*S*-Tb-Et-1** in glycerin/DMSO mixed solutions with different contents of f_w) (B, D, F, and H).
**Supplementary Figure 13**	Luminescence spectra of ***R*-Gd-Me-1** and ***R*-Tb-Me-1** in glycerin/DMSO mixtures with different f_w (A and C); Luminescence intensity of ***R*-Gd-Me-1** and ***R*-Tb-Me-1** at 443 nm as a function of f_w (B and D).
**Supplementary Figure 14**	Luminescence spectra of ***R*/*S*-Eu-Me-1** in CH_3CN/DMF mixtures with different f_w under excitation at 365 nm (A and D); Luminescence intensity of ***R*/*S*-Eu-Me-1** at 617 nm as a function of f_w (B and E); Absorption spectra of ***R*/*S*-Eu-Me-1** in CH_3CN/DMF mixed solutions with different contents of f_w, the insets show the DLS results of ***R*/*S*-Eu-Me-1** in 99% CH_3CN/DMF mixture (C and F).
**Supplementary Figure 15**	Luminescence spectra of ***S*-Eu-Et-1/2** in CH_3CN/DMF mixtures with different f_w under excitation at 365 nm (A and D); Luminescence intensity of ***S*-Eu-Et-1/2** at 617 nm as a function of f_w (B and E); Absorption spectra of ***S*-Eu-Et-1/2** in

	CH_3CN/DMF mixed solutions with different contents of f_w, the insets show the DLS results of ***S*-Eu-Et-1/2** in 99% CH_3CN/DMF mixture (C and F).
**Supplementary Figure 16**	Zeta potential of ***R*/*S*-Eu-Et-1**, ***R*/*S*-Eu-Me-1**, and ***R*/*S*-Eu-Et-2** in the mixed solvents.
**Supplementary Figure 17**	Luminescence spectra of **Eu-Et-3** in CH_3CN/DMF mixtures with different CH_3CN contents (f_w) (A); Luminescence intensity of **Eu-Et-3** at 617 nm as a function of f_w (B).
**Supplementary Figure 18**	Luminescence spectra of ***R*/*S*-Eu-R-1** (R = Et/Me) (A–D) and ***R*/*S*-Eu-Et-2** (E and F) dispersed in CH_3CN under excitation at 365 nm.
**Supplementary Figure 19**	The decay curve of $^5D_0$ energy level of ***R*/*S*-Eu-R-1** (R = Et/Me) (A, D, C, and F) and ***R*/*S*-Eu-Et-2** (B and E) dispersed in CH_3CN.
**Supplementary Figure 20**	QYs of ***R*/*S*-Eu-R-1** (R = Et/Me) (A and B) and ***R*/*S*-Eu-Et-2** (C) dispersed in CH_3CN.
**Supplementary Figure 21**	Photo of ***R*-Eu-Et-1** under CLSM (405 nm).
**Supplementary Figure 22**	CIE 1931 chromaticity diagram of the obtained red light from ***R*/*S*-Eu-R-1** (R = Et/Me) (A–D) and ***R*/*S*-Eu-Et-2** (E and F).
**Supplementary Figure 23**	Solid-state luminescence spectra ***S*-Eu-Et-1** and ***R*/*S*-Eu-Me-1** (A–C), and ***S*-Eu-Et-2** (D) under excitation at 365 nm.
**Supplementary Figure 24**	Solid-state luminescence spectra of **Eu-Et-3**.
**Supplementary Figure 25**	QYs of ***R*/*S*-Eu-R-1** (R = Et/Me) (A and B), ***R*/*S*-Eu-Et-2** (C), and **Eu-Et-3** (D).
**Supplementary Figure 26**	After turning off the 365 nm UV-light, ***R*-Eu-Et-1** (A) and ***R*-Eu-Et-2** (B) can still keep red light for a short time (Note: The iPhone splits the 1 s video into 100 pictures, each picture is 0.01 s; at the moment when the UV lamp is turned off, filter paper spots appear in the picture due to the strong afterglow of the complex. For clarity, the noise in the video has been processed using the CapCut software.).
**Supplementary Figure 27**	The decay curve of $^5D_0$ energy level in solid-state ***S*-Eu-Et-1**, ***R*/*S*-Eu-Me-1**, and ***S*-Eu-Et-2**.
**Supplementary Figure 28**	The decay curve of $^5D_0$ energy level in solid-state **Eu-Et-3**.
**Supplementary Figure 29**	Molecular ion peaks and analytical results of the HRESI-MS of *R*/*S*-L^1 (A and B) and *R*/*S*-L^2 (C and D). The fitting comparison of experimental and theoretical values of molecular ion peaks of *R*/*S*-L^1 and *R*/*S*-L^2 (E and F).
**Supplementary Figure 30**	Solid-state UV-Vis absorption spectra of ***R***-L^1 and ***R***-L^2 (A and B), Phosphorescence spectra of ***R*-Gd-Et-1/2** collected at 77 K in the solid state (C and D).
**Supplementary Figure 31**	CPL and DC spectra (A) and corresponding g_{lum} values (B) of ***R*/*S*-Eu-Me-1** dispersed in CH_3CN.
**Supplementary Figure 32**	UV-Vis spectra of ***R*/*S*-Eu-R-1** (R = Et/Me) and ***R*/*S*-Eu-Et-2** dispersed in CH_3CN.
**Supplementary Figure 33**	UV-Vis spectra of ***R*-Eu-Et-1**, ***R*-Eu-Et-1**+Cu^{II} (A), ***R*-Eu-Et-2**, and ***R*-Eu-Et-2**+Cu^{II} (B) dispersed in CH_3CN.
**Supplementary Figure 34**	Concentration-dependent spectra and fitting curves of **Eu-Et-3** on Cu^{II} ions under excitation at 365 nm.
**Supplementary Figure 35**	In the presence of another competing metal cation in CH_3CN, the change of the luminescence intensity of ***R*-Eu-Et-1** before and after the addition of Cu^{II} ions (A and B).
**Supplementary Figure 36**	Concentration-dependent spectra and fitting curves of ***R*-Eu-Et-1** (A and B) and ***R*-Eu-Et-2** (C and D) on Co^{II} ions.
**Supplementary Figure 37**	Concentration-dependent spectra and fitting curves of ***R*-Eu-Et-1** (A and B) and ***R*-Eu-Et-2** (C and D) on Fe^{III} ions.
**Supplementary Figure 38**	The fitting comparison of experimental and theoretical values of mass spectral molecular ion peaks of ***R*-Eu-Et-1** (A–D) and ***R*-Eu-Et-1** containing copper ions (E and F).
**Supplementary Figure 39**	Concentration-dependent spectra and fitting curves of ***R*-Eu-Et-1** on Cu^{II} ions under different pH conditions.
**Supplementary Figure 40**	Concentration-dependent spectra and fitting curves of ***R*-Eu-Et-2** on Cu^{II} ions under different pH conditions.

Supplementary Data	
**Supplementary Data 1**	Selected bond lengths (Å) and angles (°) of ***R*/*S*-Eu-R-1** (R = Et/Me), ***R*/*S*-Eu-Et-2**, **Eu-Et-3**, ***R*/*S*-Gd-R-1** (R = Et/Me), ***R*-Gd-Et-2**, ***R*-Tb-Et-1**, ***R*/*S*-Tb-Me-1**, and ***R*-Tb-Et-2**.
**Supplementary Data 2**	QYs of ***R*/*S*-Eu-Et-1**, ***R*/*S*-Eu-Et-1**, and ***R*/*S*-Eu-Et-2** dispersed in glycerin/DMSO or CH_3CN/DMF mixtures with different f_w.
**Supplementary Data 3**	DFT-computed coordinates of ***R*-Eu-Et-1**.
**Supplementary Data 4**	DFT-computed coordinates of ***R*-Eu-Et-2**.

Supplementary Notes

Supplementary Note 1: **Experimental Section**

Supplementary Materials and Measurements.

All reagents were obtained from commercial sources and used without further purification. Elemental analyses for C, H, and N were performed on a varia MICRO cube. Infrared spectra were recorded by transmission through KBr pellets containing *ca.* 0.5% of the complexes using a PE spectrum FT-IR spectrometer (400–4,000 cm^{-1}). Thermogravimetric analyses (TGA) were conducted in a flow of nitrogen at a heating rate of 5 °C/min using a NETZSCH TG 209 F3. UV-Vis absorption spectra were recorded on a Cary 100 UV-Vis spectrophotometer (Agilent), Shimadzu UV-2600i and UV-3600 plus, and fluorescence spectra were recorded on a Cary Eclipse fluorescence spectrophotometer (Agilent), FL970 Fluorescence Spectrophotometer (Tianmei), and Quanta Master 8000 (HORIBA CANADA). The scanning electron microscopy (SEM) images were recorded using FEI Quanta 200 Field Emission Environmental Scanning Electron Microscopy. Powder X-ray diffraction (PXRD) spectra were recorded on either a D8 Advance (Bruker) diffractometer at 293 K (Mo-Kα). The samples were prepared by crushing crystals and the powder placed on a grooved aluminum plate. Diffraction patterns were recorded from 5° to 55° at a rate of 5° min^{-1}. Circularly polarized spectra (CPL) were recorded on a Circularly Polarized Luminescence JASCO CPL-300 Spectrometer.

Supplementary Note 2: **Single-crystal X-ray crystallography**.

Diffraction data for the complex were collected on a Bruker SMART CCD diffractometer (Cu-Kα radiation and λ = 1.54 Å) in Φ and ω scan modes. The structures were solved by direct methods, followed by difference Fourier syntheses, and then refined by full-matrix least-squares techniques on F^2 using *SHELXL*[1]. All other non-hydrogen atoms were refined with anisotropic thermal parameters. Hydrogen atoms were placed at calculated positions and isotopically refined using a riding model. Supplementary Table 1 summarizes X-ray crystallographic data and refinement details for the complexes.

Supplementary Note 3: **Containing the Checkcif alerts obtained from the checkcif.iucr.org webpage and the authors responses.**

***S*-Eu-Et-1:**

PLAT241_ALERT_2_B High ’MainMol’ Ueq as Compared to Neighbors of O7 Check

Response: Some refined commands such as ISOR, DELU and SIMU were used to address such abnormal Ueq value of some O atoms but failed.

PLAT241_ALERT_2_B High ’MainMol’ Ueq as Compared to Neighbors of O8 Check

Response: Some refined commands such as ISOR, DELU and SIMU were used to address such abnormal Ueq value of some O atoms but failed.

***S*-Gd-Et-1:**

PLAT987_ALERT_1_B The Flack x is >> 0 - Do a BASF/TWIN Refinement Please Check

Response: All data fit a single domain, no twin.

Supplementary Table 1. Crystallographic data of ***R*/*S*-Eu-R-1** (R = Et/Me), ***R*/*S*-Eu-Et-2**, **Eu-Et-3**, ***R*/*S*-Gd-R-1** (R = Et/Me), ***R*-Gd-Et-2**, ***R*-Tb-Et-1**, ***R*/*S*-Tb-Me-1**, and ***R*-Tb-Et-2**.

	***R*-Eu-Et-1**	***S*-Eu-Et-1**	***R*-Eu-Me-1**	***S*-Eu-Me-1**
Formula	$(C_{26}H_{28}N_9O_9Eu)_2$	$(C_{26}H_{28}N_9O_9Eu)_2$	$(C_{24}H_{24}N_9O_9Eu)_2$	$(C_{24}H_{24}N_9O_9Eu)_2$
Formula weight	1525.06	1525.06	1468.96	1468.96
T, K	293(2)	293(2)	293(2)	293(2)
Crystal system	Orthorhombic	Orthorhombic	Orthorhombic	Orthorhombic
Space group	$P2_12_12_1$	$P2_12_12_1$	$P2_12_12_1$	$P2_12_12_1$
a, Å	14.4824(2)	21.4141(4)	14.3112(2)	14.3106(2)
b, Å	21.3927(4)	19.7954(3)	19.2922(3)	19.2867(2)
c, Å	19.7825(2)	14.4850(2)	20.9967(3)	21.0081(4)
α, °	90	90	90	90
β, °	90	90	90	90
γ, °	90	90	90	90
V, Å^3	6128.97(16)	6140.20(17)	5797.08(15)	5798.33(15)
Z	4	4	4	4
D_c, g·cm^{-3}	1.653	1.650	1.683	1.683
μ, mm^{-1}	2.113	2.109	2.23	2.230
F(000)	3056	3056	2928	2928
2θ range for data collection/°	6.694 to 57.784	6.786 to 57.912	6.626 to 57.454	6.628 to 57.62
Reflns coll.	49853	26228	27181	24635
Unique reflns	14333	13709	13201	12565
R_{int}	0.0334	0.0290	0.0239	0.0269
Observed data [I> 2σ(I)]	12041	11101	11160	10608
N_{par}, N_{ref}	815, 14333	815, 13709	779, 13201	779, 12565
R_1[a] (I> 2σ(I))	0.0371	0.0409	0.0330	0.0338
wR_2[b] (all data)	0.0924	0.1009	0.0744	0.0716
GOF	1.069	1.064	1.058	1.053
CCDC	2262497	2262500	2262499	2262503

[a]$R_1=\Sigma||F_o|-|F_c||/\Sigma|F_o|$, [b]$wR_2 = [\Sigma w(F_o^2-F_c^2)^2/\Sigma w(F_o^2)^2]^{1/2}$

	***R*-Eu-Et-2**	***S*-Eu-Et-2**	**Eu-Et-3**	***R*-Gd-Et-1**
Formula	$C_{20}H_{29}N_{10}O_9Eu$	$C_{20}H_{29}N_{10}O_9Eu$	$C_{14}H_{20}EuN_9O_9$	$(C_{26}H_{28}N_9O_9Gd)_2$
Formula weight	705.49	705.49	610.35	1535.64
T, K	100(2)	100(2)	100(2)	293(2)
Crystal system	Orthorhombic	Orthorhombic	Triclinic	Orthorhombic
Space group	$P2_12_12_1$	$P2_12_12_1$	$P\bar{1}$	$P2_12_12_1$
a, Å	8.37480(10)	8.3866(1)	8.35333(15)	21.3519(3)
b, Å	17.3275(2)	17.3442(2)	9.9825(2)	19.7749(3)
c, Å	19.1939(2)	19.1917(2)	14.51363(19)	14.4701(2)
α, °	90	90	99.4865(15)	90
β, °	90	90	102.2098(13)	90
γ, °	90	90	108.2188(18)	90
V, Å^3	2786.17(5)	2791.60(5)	1088.26(4)	6109.73(15)
Z	4	4	2	4
D_c, g·cm^{-3}	1.682	1.679	1.863	1.669
μ, mm^{-1}	16.699	16.662	21.239	2.24
F(000)	1416.0	1416.0	604.0	3064

2θ range for data collection/°	6.872 to 156.494	6.87 to 151.286	6.434 to 146.53	6.792 to 57.926
Reflns coll.	13589	10413	13980	26319
Unique reflns	5121	5265	4222	12930
R_{int}	0.0491	0.0574	0.0388	0.025
Observed data [$I > 2\sigma(I)$]	4974	5023	4077	10701
N_{par}, N_{ref}	364,5121	364,5265	301,4222	390, 815
R_1^a ($I > 2\sigma(I)$)	0.0395	0.0518	0.0621	0.0371
wR_2^b (all data)	0.1103	0.1572	0.0627	0.0890
GOF	0.977	1.163	1.042	1.068
CCDC	2262498	2262502	2307112	2308025

$^aR_1=\Sigma||F_o|-|F_c||/\Sigma|F_o|$, $^bwR_2 = [\Sigma w(F_o^2-F_c^2)^2/\Sigma w(F_o^2)^2]^{1/2}$

	***S*-Gd-Et-1**	***R*-Gd-Me-1**	***S*-Gd-Me-1**	***R*-Gd-Et-2**
Formula	$(C_{26}H_{28}N_9O_9Gd)_2$	$(C_{24}H_{24}N_9O_9Gd)_2$	$(C_{24}H_{24}N_9O_9Gd)_2$	$C_{20}H_{29}GdN_{10}O_9$
Formula weight	1535.64	1479.54	1479.54	710.78
T, K	293(2)	293(2)	293(2)	100
Crystal system	Orthorhombic	Orthorhombic	Orthorhombic	Orthorhombic
Space group	$P2_12_12_1$	$P2_12_12_1$	$P2_12_12_1$	$P2_12_12_1$
a, Å	14.47585(7)	14.3381(3)	14.3030(2)	8.3892(2)
b, Å	21.37867(9)	19.2798(5)	19.2836(3)	17.3155(3)
c, Å	19.78454(9)	20.9991(6)	20.9871(3)	19.1708(4)
α, °	90	90	90	90
β, °	90	90	90	90
γ, °	90	90	90	90
V, Å^3	6122.81(5)	5804.9(3)	5788.52(15)	2784.81(10)
Z	4	4	4	4
D_c, g·cm^{-3}	1.666	1.693	1.698	1.695
μ, mm^{-1}	14.59	2.35	2.36	15.98
F(000)	3064	2936	2936	1420
2θ range for data collection/°	6.086 to 149.06	6.814 to 57.458	6.82 to 57.93	6.878 to 145.192
Reflns coll.	45841	24571	26605	10758
Unique reflns	12049	12515	13090	4730
R_{int}	0.055	0.027	0.0282	0.034
Observed data [$I > 2\sigma(I)$]	11162	10524	10932	4612
N_{par}, N_{ref}	390, 816	779, 12515	779, 13090	365, 4730
R_1^a ($I > 2\sigma(I)$)	0.0469	0.0326	0.0338	0.0306
wR_2^b (all data)	0.1321	0.0729	0.0724	0.0778
GOF	1.050	1.054	1.065	1.058
CCDC	2308037	2308027	2308038	2308026

$^aR_1=\Sigma||F_o|-|F_c||/\Sigma|F_o|$, $^bwR_2 = [\Sigma w(F_o^2-F_c^2)^2/\Sigma w(F_o^2)^2]^{1/2}$

	***R*-Tb-Et-1**	***R*-Tb-Me-1**	***S*-Tb-Me-1**	***R*-Tb-Et-2**
Formula	$(C_{26}H_{28}N_9O_9Tb)_2$	$(C_{24}H_{24}N_9O_9Tb)_2$	$(C_{24}H_{24}N_9O_9Tb)_2$	$C_{20}H_{29}N_{10}O_9Tb$
Formula weight	1538.98	1482.88	1482.88	712.45
T, K	293(2)	293(2)	293(2)	100
Crystal system	Orthorhombic	Orthorhombic	Orthorhombic	Orthorhombic
Space group	$P2_12_12_1$	$P2_12_12_1$	$P2_12_12_1$	$P2_12_12_1$

a, Å	21.3612(3)	19.2582(4)	14.3051(2)	8.38315(13)
b, Å	19.7621(2)	14.3119(3)	19.2678(2)	17.3227(3)
c, Å	14.4631(2)	20.9476(5)	20.9928(3)	19.1459(3)
α, °	90	90	90	90
β, °	90	90	90	90
γ, °	90	90	90	90
V, Å^3	6105.48(14)	5773.6(2)	5786.20(13)	2780.34(8)
Z	4	4	4	4
D_c, g·cm^{-3}	1.674	1.706	1.702	1.702
μ, mm^{-1}	2.38	2.52	2.51	13.09
F(000)	3072	2944	2944	1424
2θ range for data collection/°	6.796 to 57.84	6.828 to 57.758	6.634 to 57.504	6.882 to 146.02
Reflns coll.	27472	25074	24863	10629
Unique reflns	13588	12792	12895	4878
R_{int}	0.028	0.0377	0.025	0.038
Observed data [$I > 2\sigma(I)$]	10972	10517	10946	4750
N_{par}, N_{ref}	815, 13588	779, 12792	779, 12895	364, 4878
R_1[a] ($I > 2\sigma(I)$)	0.0417	0.0377	0.0317	0.0387
wR_2[b] (all data)	0.1086	0.0771	0.0672	0.1073
GOF	1.056	1.070	1.056	1.096
CCDC	2308028	2308033	2308040	2308029

[a]$R_1 = \Sigma||F_o|-|F_c||/\Sigma|F_o|$, [b]$wR_2 = [\Sigma w(F_o^2-F_c^2)^2/\Sigma w(F_o^2)^2]^{1/2}$

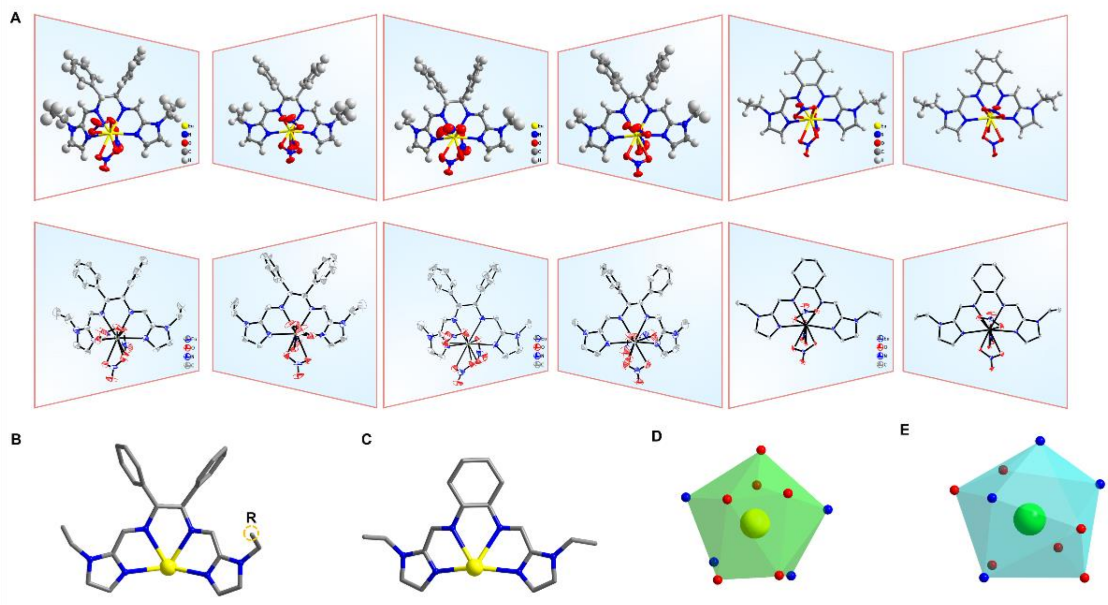

Supplementary Figure 1. **Structural analysis**. Probabilistic ellipsoids and ORTEP-style crystal structure of ***R***/***S***-**Eu-R-1** (R = Et/Me) and ***R***/***S***-**Eu-Et-2** (A), Ligand coordinated modes and coordinated polyhedron around the metal ions of ***R***/***S***-**Eu-R-1** (R = Et/Me) and ***R***/***S***-**Eu-Et-2** (B–E).

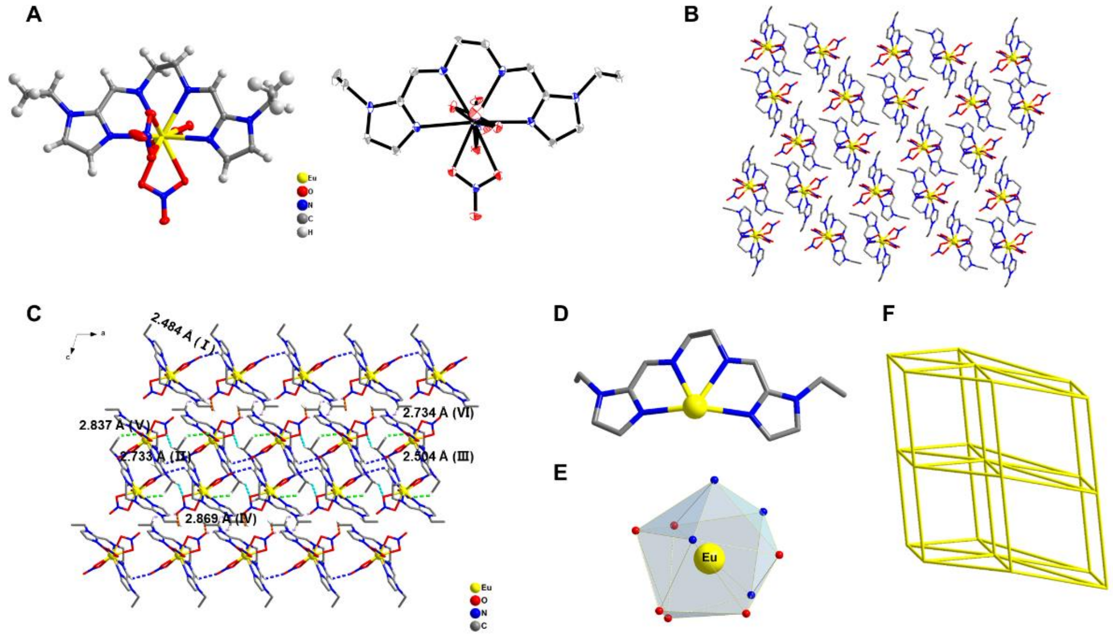

Supplementary Figure 2. **Structural analysis**. Probabilistic ellipsoids and ORTEP-style crystal structure of **Eu-Et-3** (A), Molecular stacking diagram of **Eu-Et-3** (B), Hydrogen bonding of **Eu-Et-3** (C), Ligand coordinated modes (D), Coordinated polyhedron around the metal ions of **Eu-Et-3** (E), Topological structure of **Eu-Et-3** (F).

Supplementary Notes 4: **FTIR characterizations**

R/***S***-**Eu-R-1** (R = Et/Me) and ***R***/***S***-**Eu-Et-2**, which are enantiomers, have the same Fourier transform infrared characteristic absorption peak (FTIR) at the similar position (Supplementary Figure

3). The broad absorption peak near 3400 cm^{-1} in their IR spectra can be attributed to the stretching vibration of *v*(N–H) in the imidazole ring. The strong peak around 1620 cm^{-1} can be attributed to the stretching vibration of the C=N bond of the imine group (–C=N–). The strong absorption peaks around 1480 and 1290 cm^{-1} can be attributed to the stretching vibrations of C=N and C=C bonds in the aromatic ring, respectively.

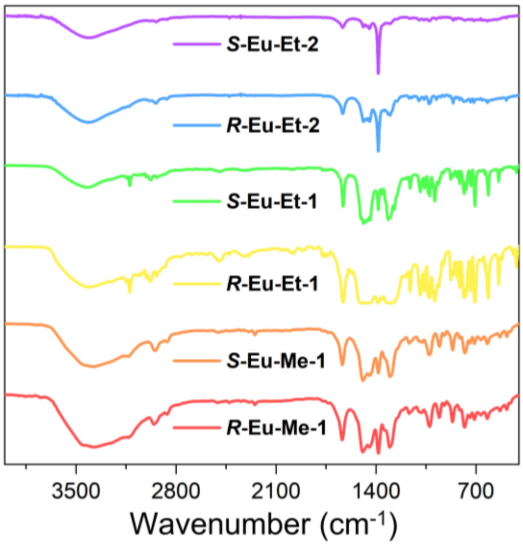

Supplementary Figure 3. IR spectra of ***R*/*S*-Eu-R-1** (R = Et/Me) and ***R*/*S*-Eu-Et-2**.

Supplementary Notes 5: **TG characterizations**

The thermal stability tests of ***R*/*S*-Eu-R-1** (R = Et/Me) and ***R*/*S*-Eu-Et-2** were carried out under a flowing nitrogen atmosphere, and the temperature was slowly increased from 35 °C to 1000 °C at a rate of 5 °C min^{-1}. ***R*/*S*-Eu-R-1** (R = Et/Me) remained stable until 255 °C without significant weight loss. ***R*/*S*-Eu-R-1** was decomposed as the temperature continued to rise (Supplementary Figure 4A–D). The weight loss of ***R*/*S*-Eu-Et-2** was 5.83% before 113 °C, which corresponds to the loss of one free CH_3CN solvent molecule (theoretical value is 5.81%). As the temperature continues to rise to 205 °C, this corresponds to the loss of one nitrate ion (theoretical value is 5.43%). ***R*/*S*-Eu-Et-2** was decomposed when the temperature continued to rise (Supplementary Figure 4E–F).

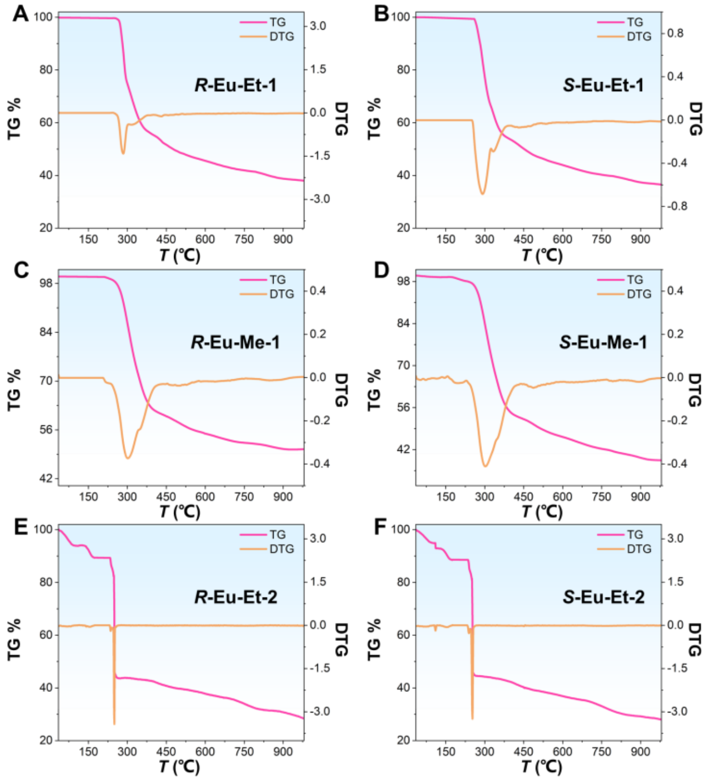

Supplementary Figure 4. **TG characterizations**. Thermogravimetric curves of ***R*/*S*-Eu-R-1** (R = Et/Me) (A–D) and ***R*/*S*-Eu-Et-2** (E and F).

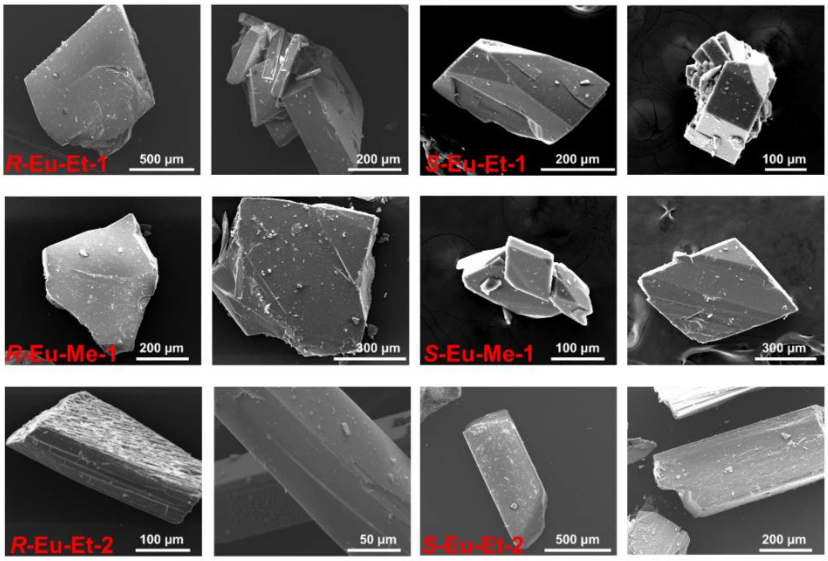

Supplementary Figure 5. SEM images of ***R*/*S*-Eu-R-1** (R = Et/Me) and ***R*/*S*-Eu-Et-2**.

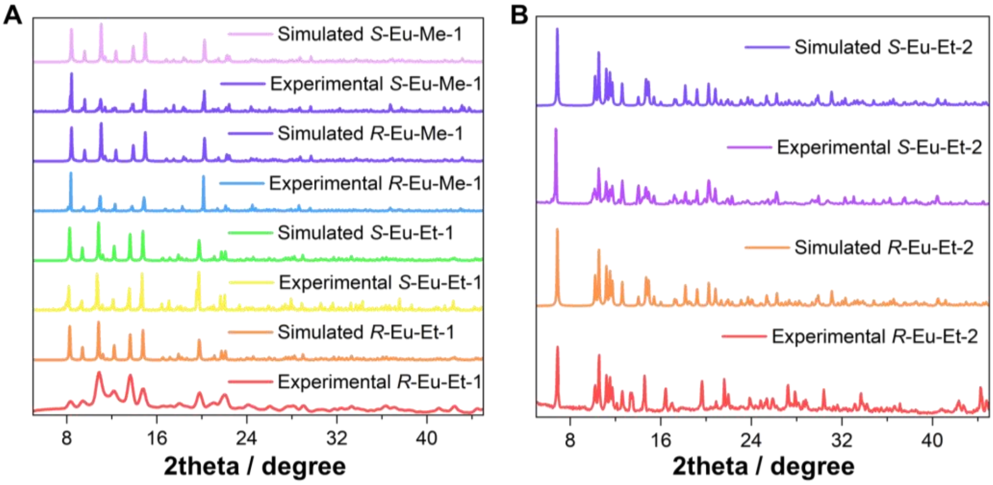

Supplementary Figure 6. **XRD characterizations**. Powder X-ray diffraction patterns (PXRD) of ***R***/***S*-Eu-R-1** (R = Et/Me) (A) and ***R***/***S*-Eu-Et-2** (B).

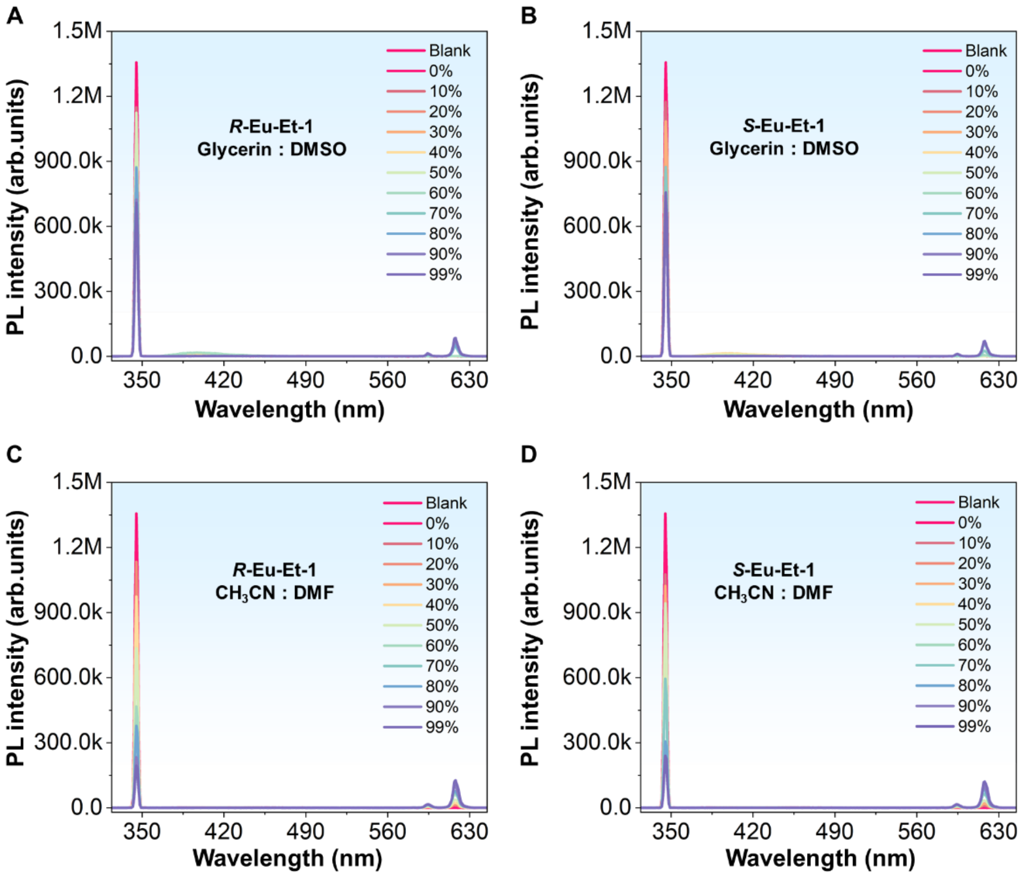

Supplementary Figure 7. **QYs characterizations**. QYs of ***R***/***S*-Eu-Et-1** dispersed in glycerin/DMSO (A and B) or CH_3CN/DMF (C and D) mixtures with different f_w.

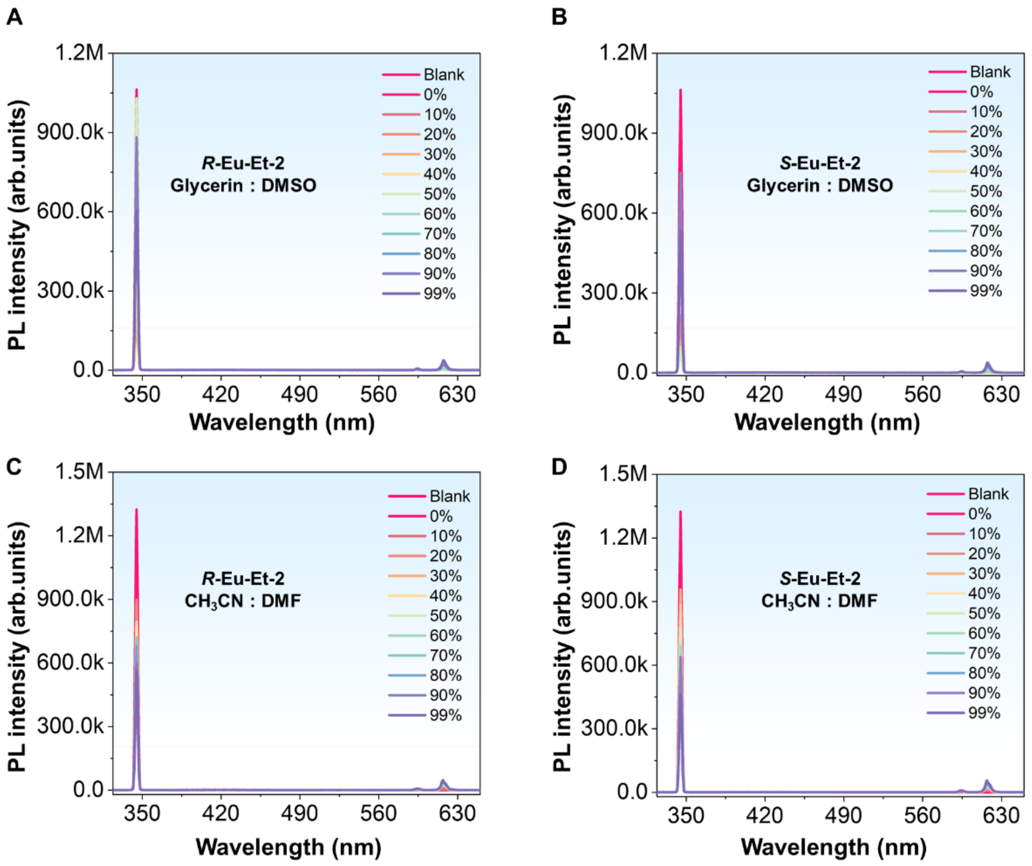

Supplementary Figure 8. **QYs characterizations**. QYs of ***R*/*S*-Eu-Et-2** dispersed in glycerin/DMSO (A and B) or CH_3CN/DMF (C and D) mixtures with different f_w.

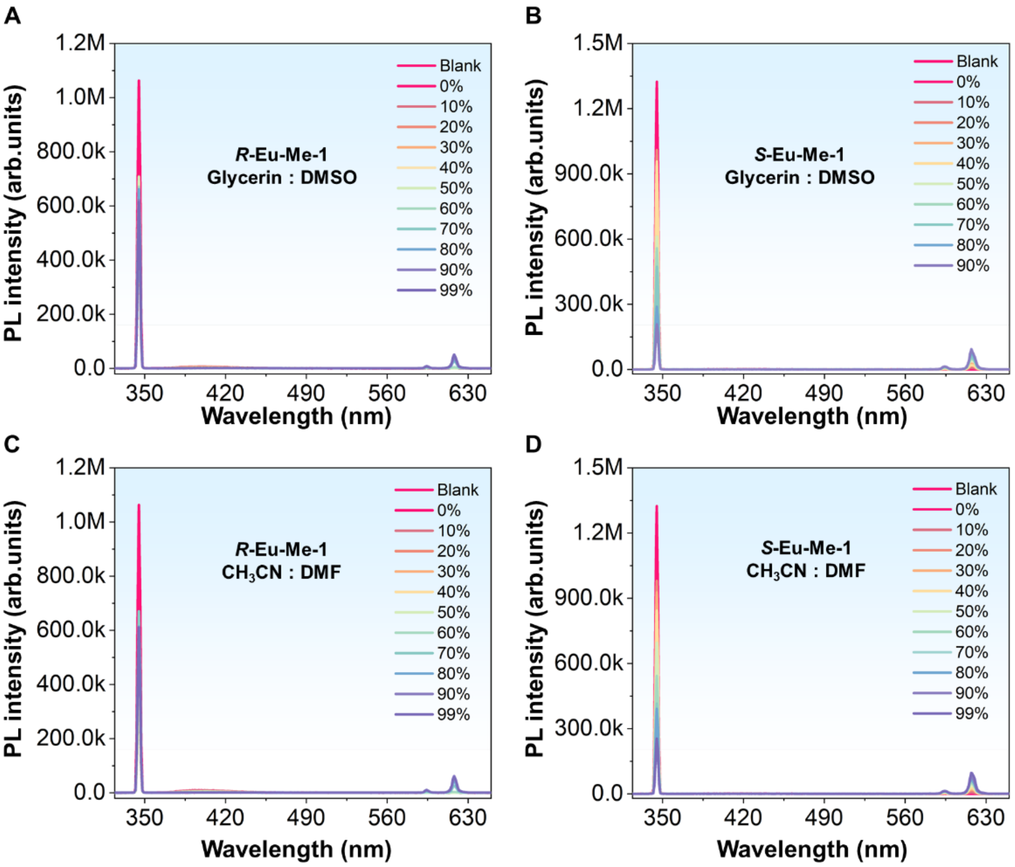

Supplementary Figure 9. **QYs characterizations**. QYs of ***R***/***S*-Eu-Me-1** dispersed in glycerin/DMSO (A and B) or CH_3CN/DMF (C and D) mixtures with different f_w.

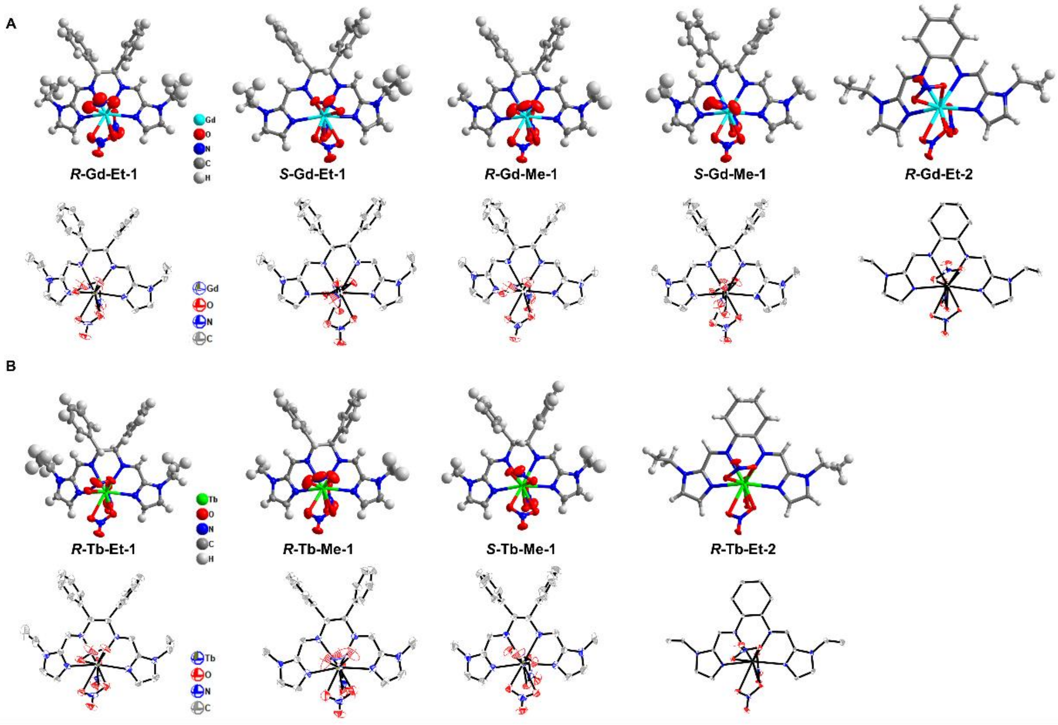

Supplementary Figure 10. **Structural analysis**. Molecular structures and ORTEP-style crystal structure of ***R*-Gd-R-1** (R = Et/Me) and ***R*-Gd-Et-2** (A) and ***R*-Tb-R-1** and ***R*-Tb-Et-2** (B).

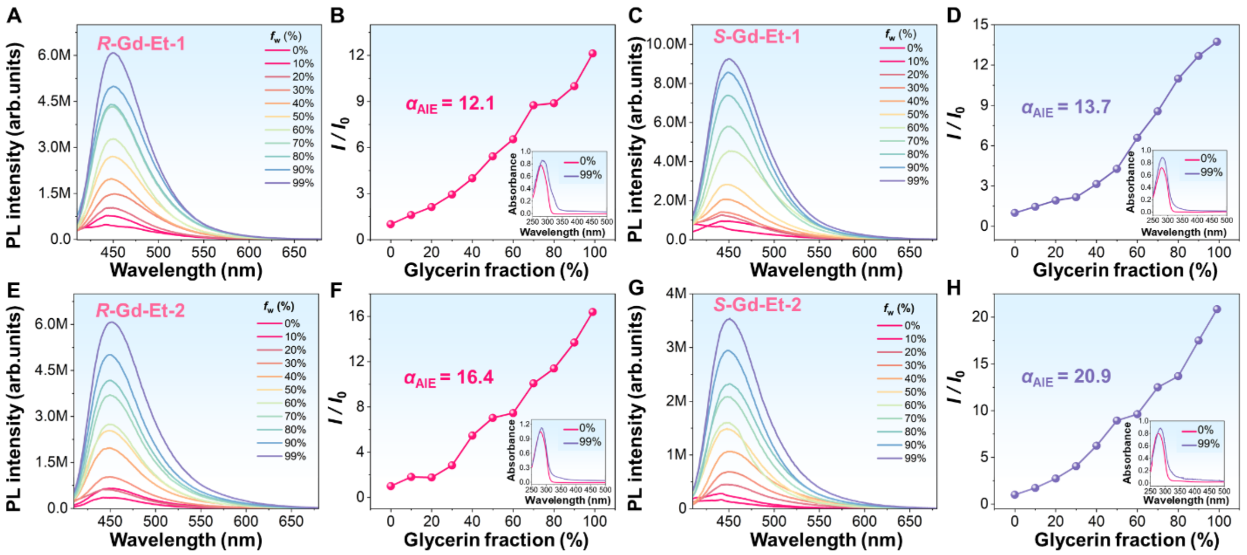

Supplementary Figure 11. **AIE performance characterization**. Luminescence spectra of ***R***/***S*-Gd-Et-1** and ***R***/***S*-Gd-Et-2** in glycerin/DMSO mixtures with different f_W (A, C, E, and G); Luminescence intensity of ***R***/***S*-Gd-Et-1** and ***R***/***S*-Gd-Et-2** at 443 nm as a function of f_W (the inset shows that the absorption spectra of ***R***/***S*-Gd-Et-1** and ***R***/***S*-Gd-Et-2** in glycerin/DMSO mixed solutions with different contents of f_W) (B, D, F, and H).

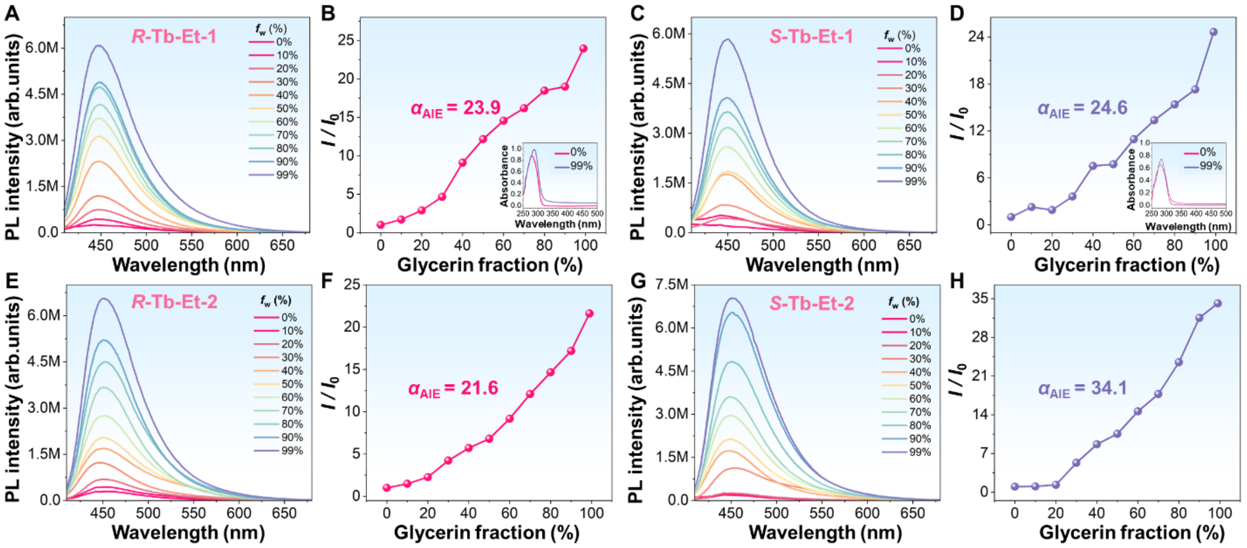

Supplementary Figure 12. **AIE performance characterization**. Luminescence spectra of ***R***/***S*-Tb-Et-1** and ***R***/***S*-Tb-Et-2** in glycerin/DMSO mixtures with different f_W (A, C, E, and G); Luminescence intensity of ***R***/***S*-Tb-Et-1** and ***R***/***S*-Tb-Et-2** at 443 nm as a function of f_W (the inset shows that the absorption spectra of ***R***/***S*-Tb-Et-1** in glycerin/DMSO mixed solutions with different contents of f_W) (B, D, F, and H).

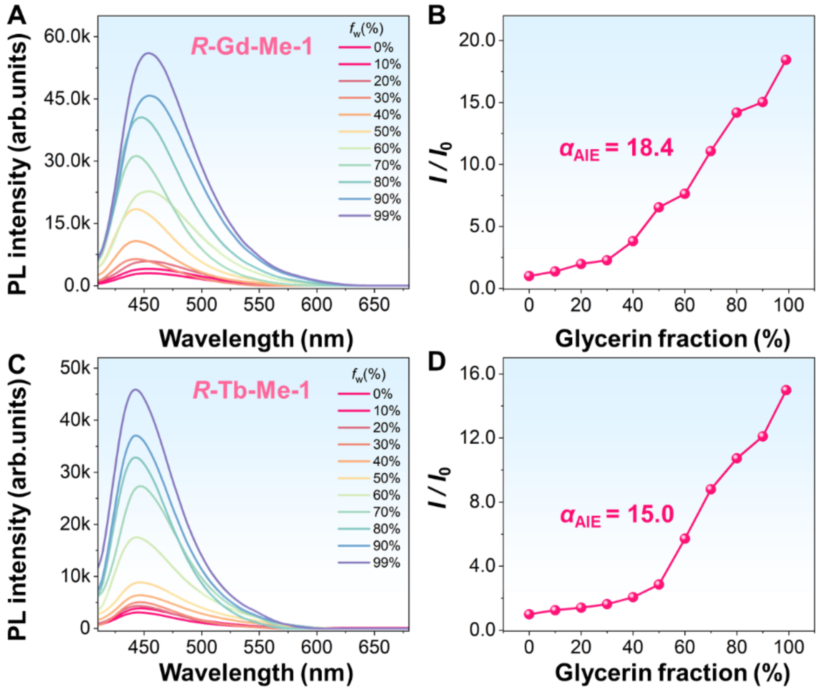

Supplementary Figure 13. **AIE performance characterization**. Luminescence spectra of ***R*-Gd-Me-1** and ***R*-Tb-Me-1** in glycerin/DMSO mixtures with different f_w (A and C); Luminescence intensity of ***R*-Gd-Me-1** and ***R*-Tb-Me-1** at 443 nm as a function of f_w (B and D).

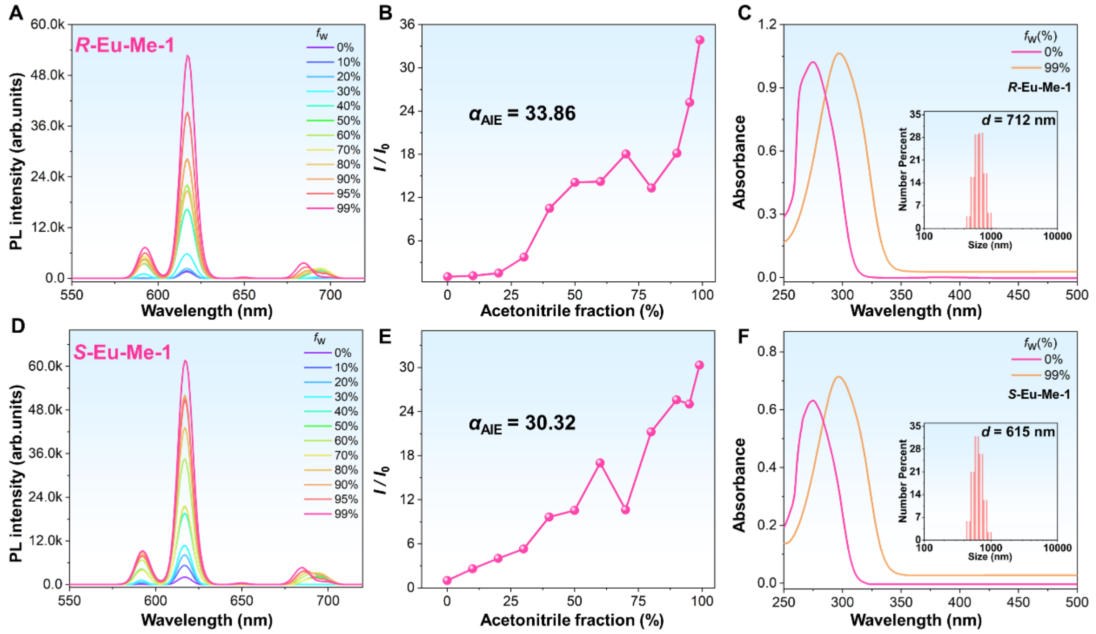

Supplementary Figure 14. **AIE performance characterization**. Luminescence spectra of ***R*/*S*-Eu-Me-1** in CH_3CN/DMF mixtures with different f_w under excitation at 365 nm (A and D); Luminescence intensity of ***R*/*S*-Eu-Me-1** at 617 nm as a function of f_w (B and E); Absorption spectra of ***R*/*S*-Eu-Me-1** in CH_3CN/DMF mixed solutions with different contents of f_w, the insets show the DLS results of ***R*/*S*-Eu-Me-1** in 99% CH_3CN/DMF mixture (C and F).

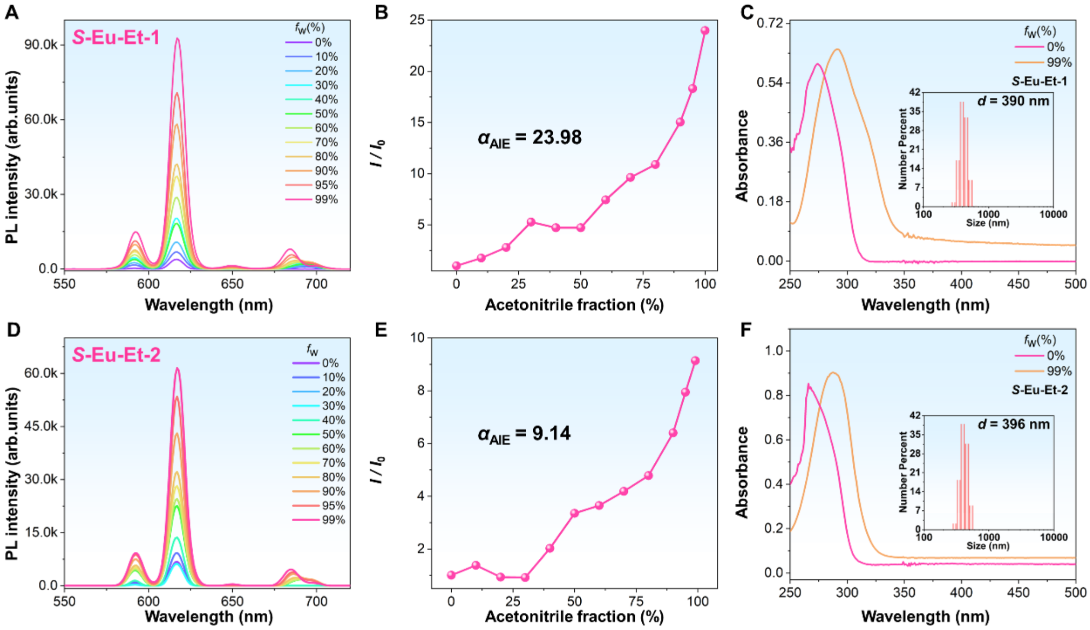

Supplementary Figure 15. **AIE performance characterization**. Luminescence spectra of ***S*-Eu-Et-1**/**2** in CH_3CN/DMF mixtures with different f_w under excitation at 365 nm (A and D); Luminescence intensity of ***S*-Eu-Et-1**/**2** at 617 nm as a function of f_w (B and E); Absorption spectra of ***S*-Eu-Et-1**/**2** in CH_3CN/DMF mixed solutions with different contents of f_w, the insets show the DLS results of ***S*-Eu-Et-1**/**2** in 99% CH_3CN/DMF mixture (C and F).

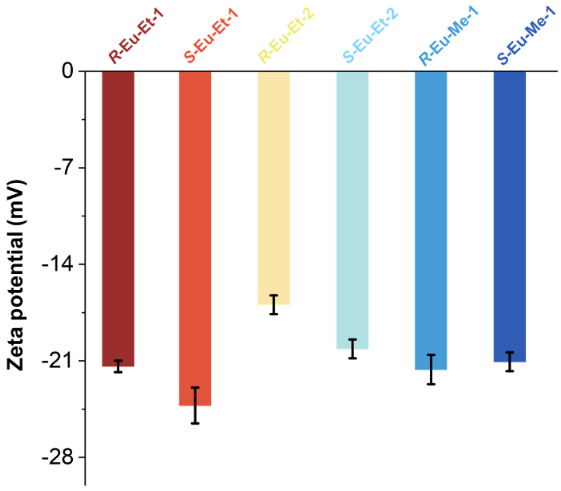

Supplementary Figure 16. Zeta potential of ***R*/*S*-Eu-Et-1**, ***R*/*S*-Eu-Me-1**, and ***R*/*S*-Eu-Et-2** in the mixed solvents.

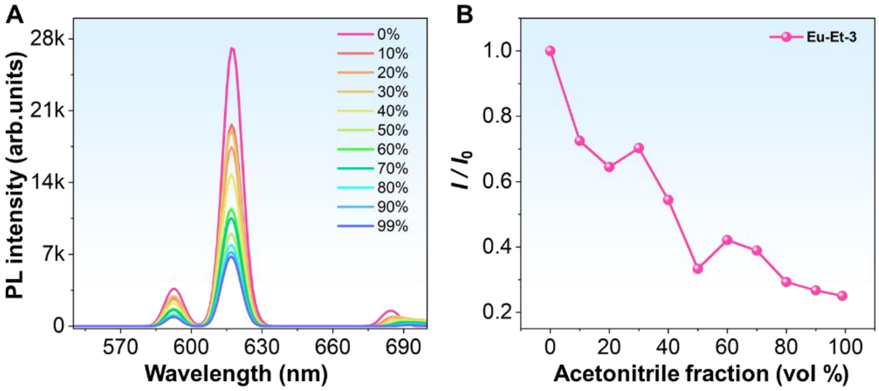

Supplementary Figure 17. **Titration curve characterization**. Luminescence spectra of **Eu-Et-3** in CH_3CN/DMF mixtures with different CH_3CN contents (f_w) (A); Luminescence intensity of **Eu-Et-3** at 617 nm as a function of f_w (B).

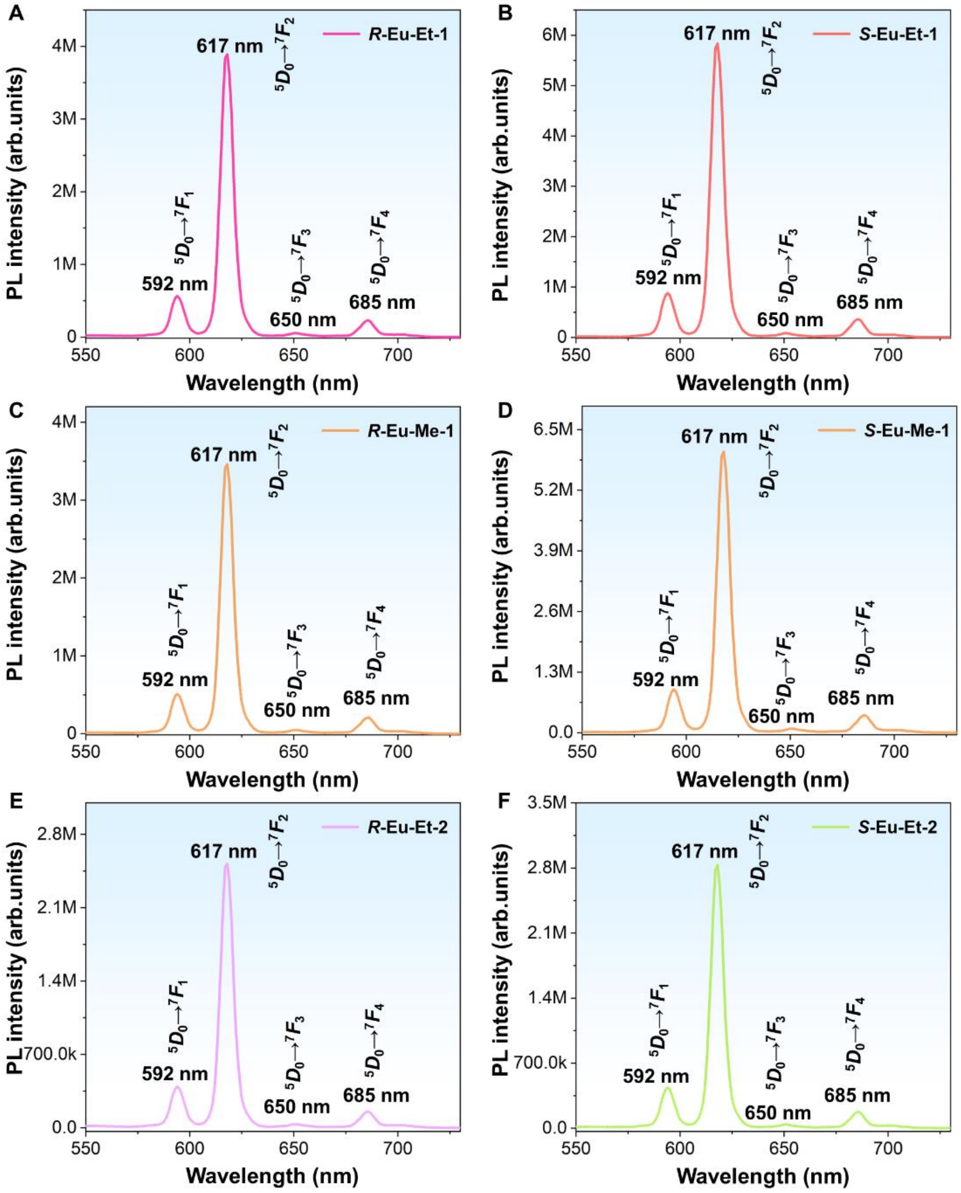

Supplementary Figure 18. **Luminescence performance characterization**. Luminescence spectras of ***R*/*S*-Eu-R-1** (R = Et/Me) (A–D) and ***R*/*S*-Eu-Et-2** (E and F) dispersed in CH_3CN under excitation at 365 nm.

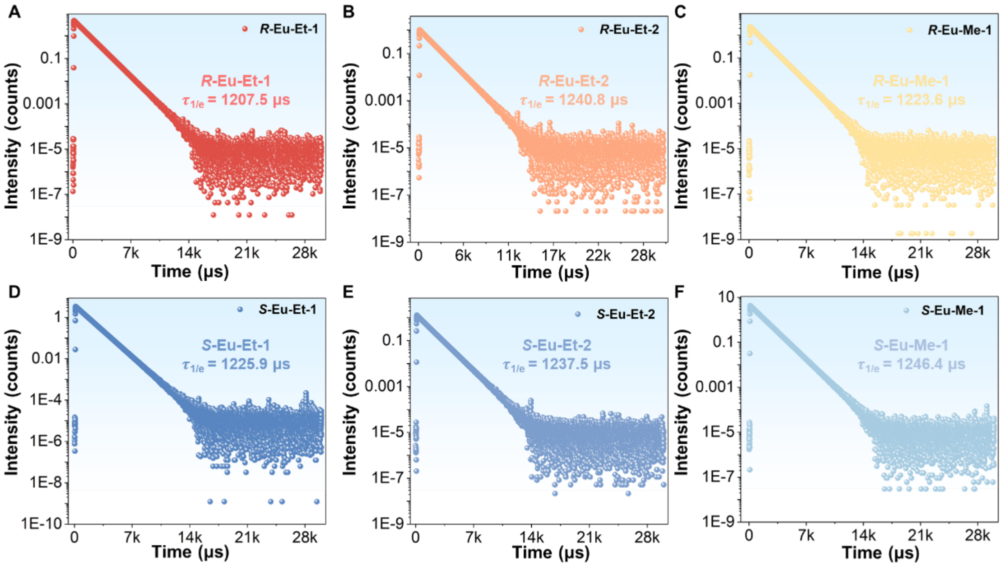

Supplementary Figure 19. **Luminescence lifetimes characterization**. The decay curve of $^5D_0$ energy level of ***R*/*S*-Eu-R-1** (R = Et/Me) (A, D, C, and F) and ***R*/*S*-Eu-Et-2** (B and E) dispersed in CH_3CN.

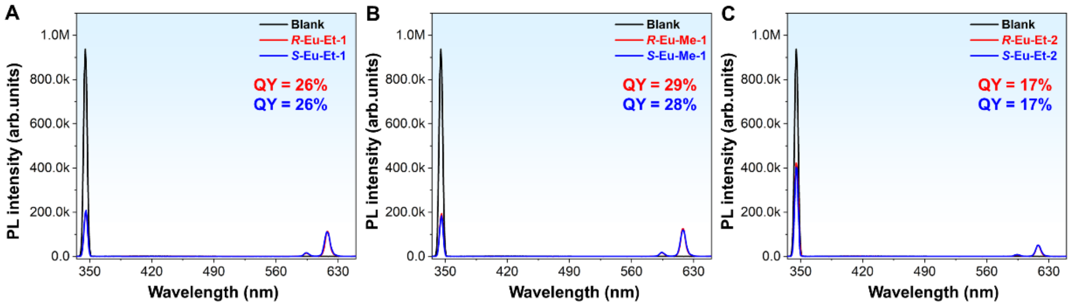

Supplementary Figure 20. **QYs characterization**. QYs of ***R*/*S*-Eu-R-1** (R = Et/Me) (A and B) and ***R*/*S*-Eu-Et-2** (C) dispersed in CH_3CN.

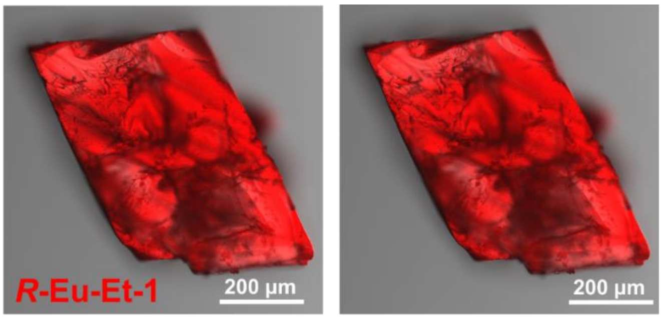

Supplementary Figure 21. Photo of ***R*-Eu-Et-1** under CLSM (405 nm).

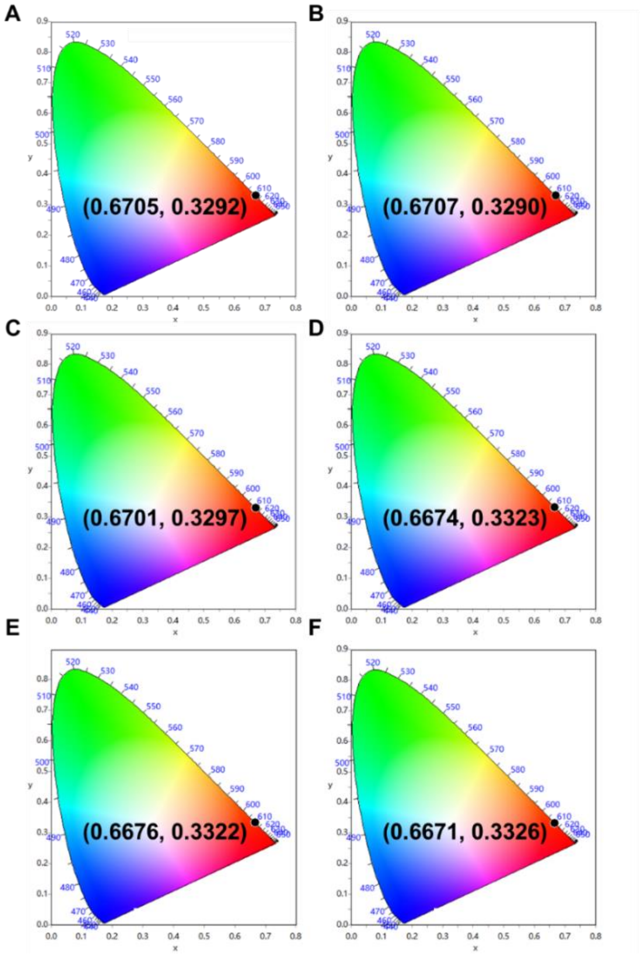

Supplementary Figure 22. **CIE view**. CIE 1931 chromaticity diagram of the obtained red light from ***R*/*S*-Eu-R-1** (R = Et/Me) (A–D) and ***R*/*S*-Eu-Et-2** (E and F).

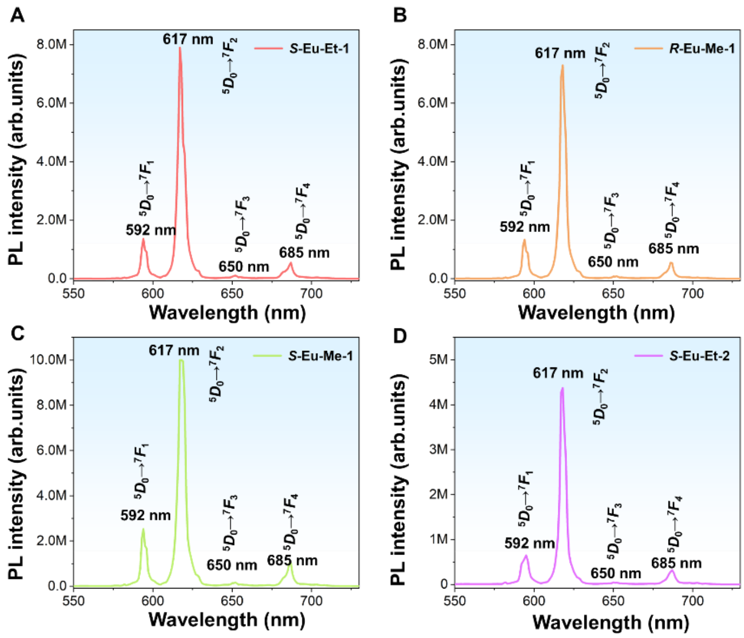

Supplementary Figure 23. **Luminescence performance characterization**. Solid-state luminescence spectra ***S*-Eu-Et-1** and ***R*/*S*-Eu-Me-1** (A–C), and ***S*-Eu-Et-2** (D) under excitation at 365 nm.

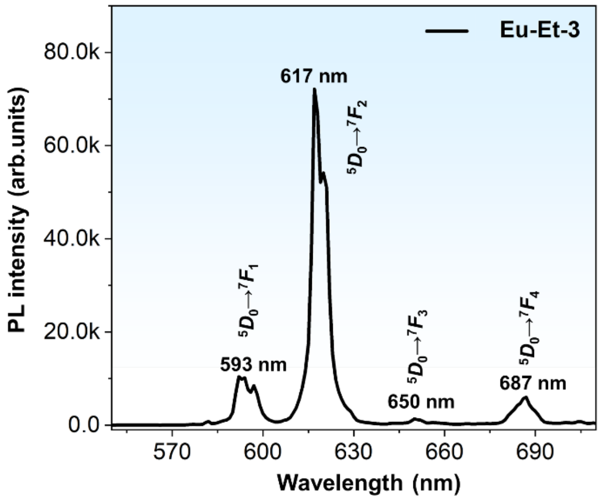

Supplementary Figure 24. Solid-state luminescence spectra of **Eu-Et-3**.

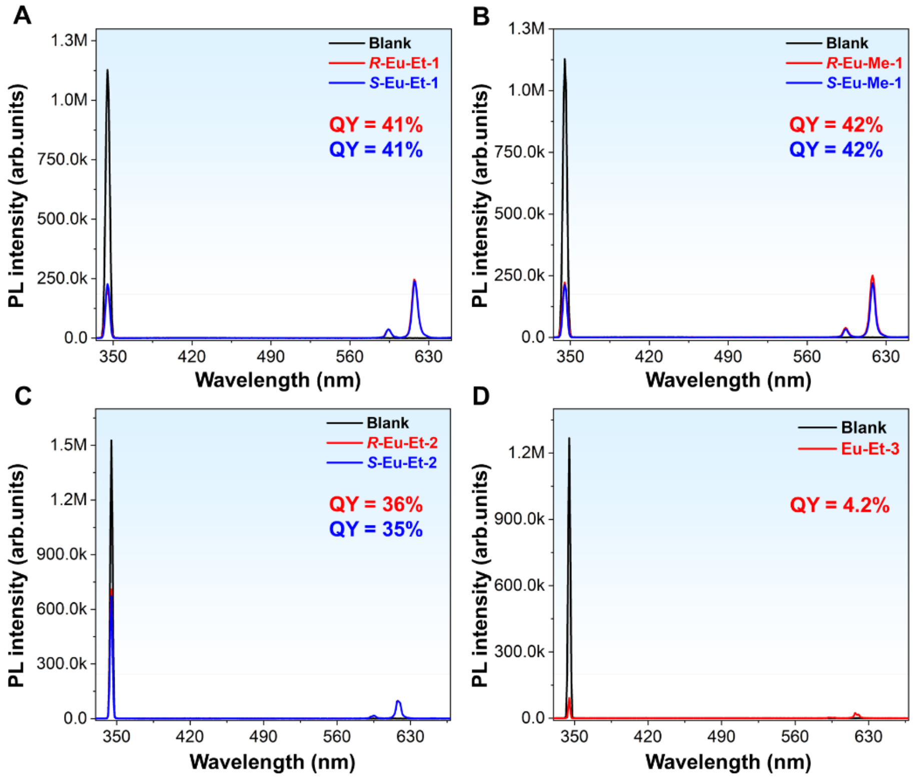

Supplementary Figure 25. **QYs characterization**. QYs of ***R***/***S*****-Eu-R-1** (R = Et/Me) (A and B), ***R***/***S*****-Eu-Et-2** (C), and **Eu-Et-3** (D).

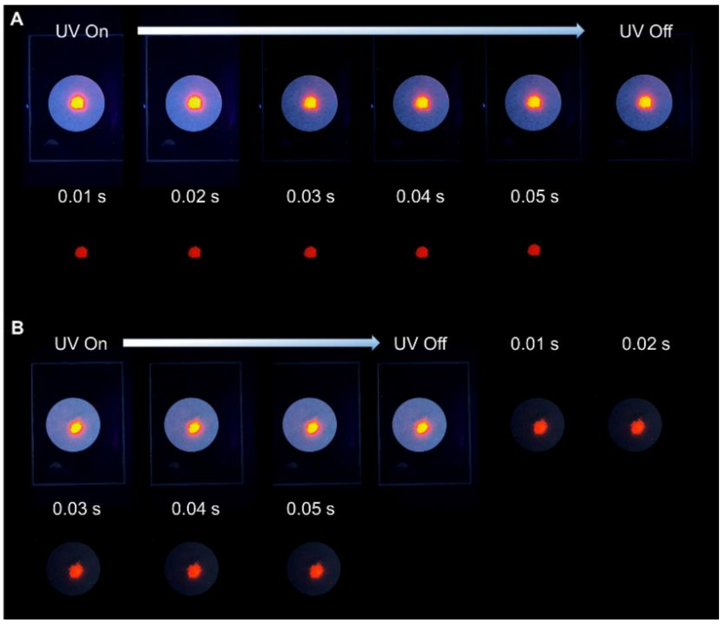

Supplementary Figure 26. After turning off the 365 nm UV-light, ***R*-Eu-Et-1** (A) and ***R*-Eu-Et-2** (B) can still keep red light for a short time (Note: The iPhone splits the 1 s video into 100 pictures, each picture is 0.01 s; at the moment when the UV lamp is turned off, filter paper spots appear in the picture due to the strong afterglow of the complex. For clarity, the noise in the video has been processed using the CapCut software.).

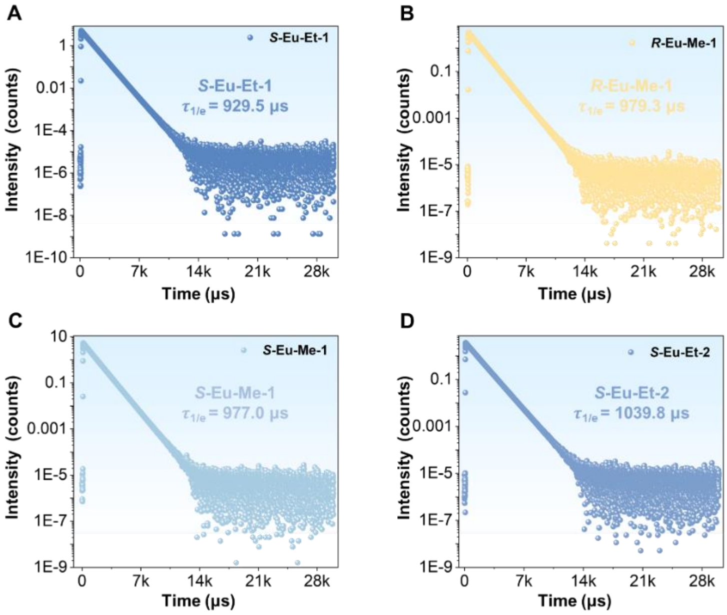

Supplementary Figure 27. **Luminescence lifetimes characterization**. The decay curve of $^5D_0$ energy level in solid-state ***S*-Eu-Et-1**, ***R*/*S*-Eu-Me-1**, and ***S*-Eu-Et-2**.

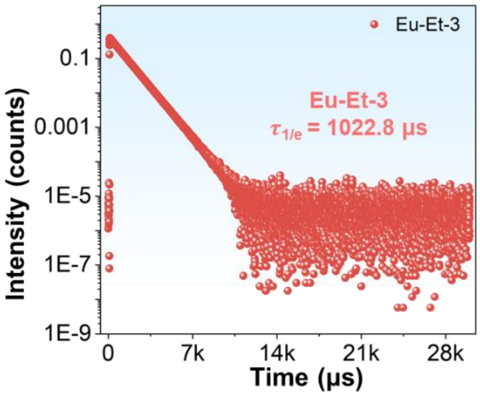

Supplementary Figure 28. The decay curve of ${}^5D_0$ energy level in solid-state **Eu-Et-3**.

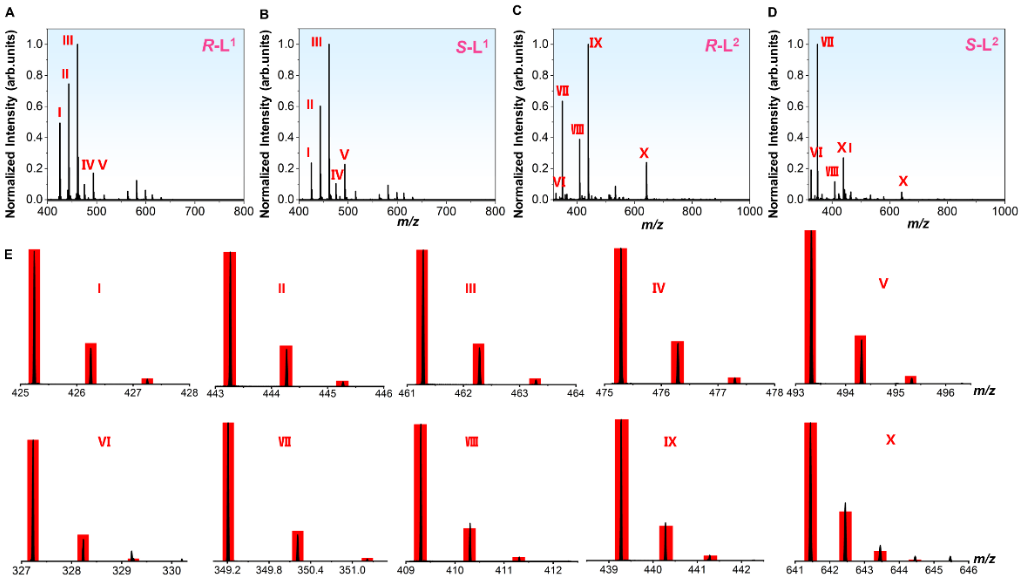

Supplementary Figure 29. **HRESI-MS of characterization.** Molecular ion peaks and analytical results of the HRESI-MS of *R*/*S*-L^1 (A and B) and *R/S*-L^2 (C and D). The fitting comparison of experimental and theoretical values of molecular ion peaks of *R*/*S*-L^1 and *R*/*S*-L^2 (E and F).

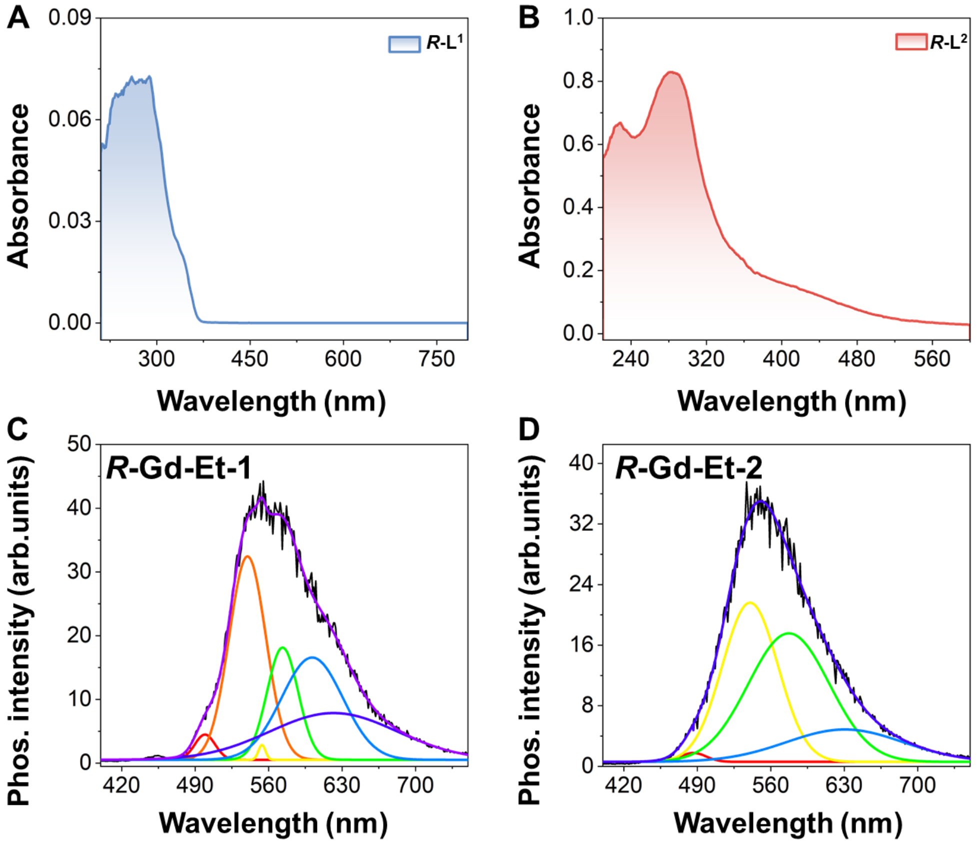

Supplementary Figure 30. **Characterization of photophysical properties**. Solid-state UV-Vis absorption spectra of ***R***-L^{1}and ***R***-L^2 (A and B); Phosphorescence spectra of ***R*-Gd-Et-1/2** collected at 77 K in the solid state (C and D).

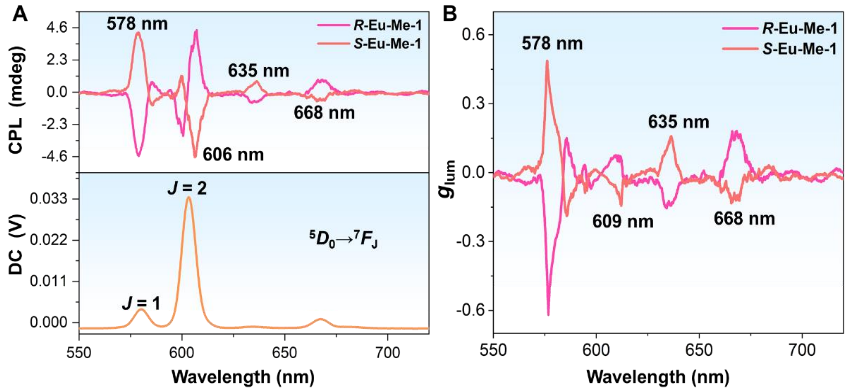

Supplementary Figure 31. **CPL and DC characterization**. CPL and DC spectra (A) and corresponding g_{lum} values (B) of ***R***/***S*-Eu-Me-1** dispersed in CH_3CN.

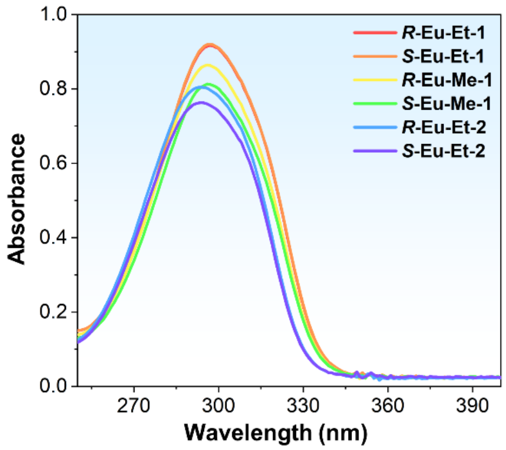

Supplementary Figure 32. UV-Vis spectra of ***R*/*S*-Eu-R-1** (R = Et/Me) and ***R*/*S*-Eu-Et-2** dispersed in CH_3CN.

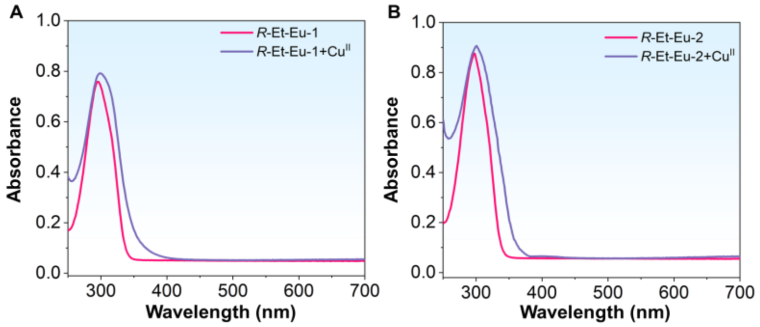

Supplementary Figure 33. **UV-Vis characterization**. UV-Vis spectra of ***R*-Eu-Et-1**, ***R*-Eu-Et-1**+Cu^{II} (A), ***R*-Eu-Et-2**, and ***R*-Eu-Et-2**+Cu^{II} (B) dispersed in CH_3CN.

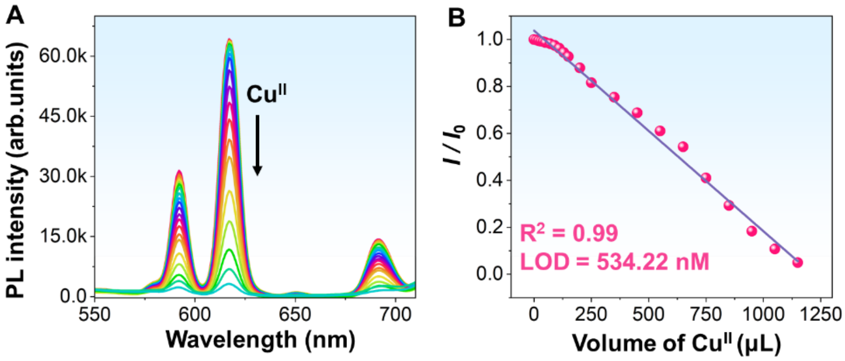

Supplementary Figure 34. **Sensing performance characterization**. Concentration-dependent spectra and fitting curves of **Eu-Et-3** on Cu^{II} ions under excitation at 365 nm.

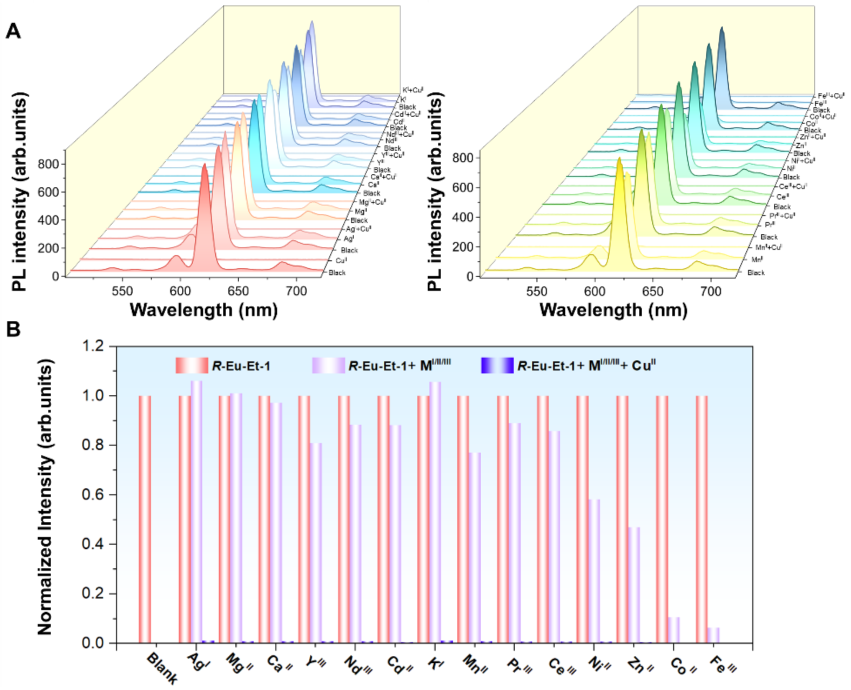

Supplementary Figure 35. In the presence of another competing metal cation in CH_3CN, the change of the luminescence intensity of ***R*-Eu-Et-1** before and after the addition of Cu^{II} ions (A and B).

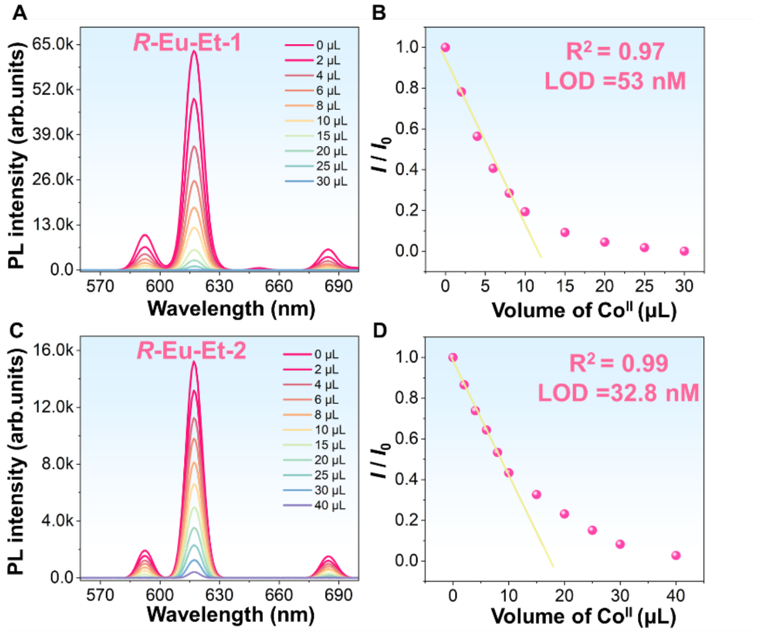

Supplementary Figure 36. **Sensing performance characterization**. Concentration-dependent spectra and fitting curves of ***R*-Eu-Et-1** (A and B) and ***R*-Eu-Et-2** (C and D) on Co^{II} ions.

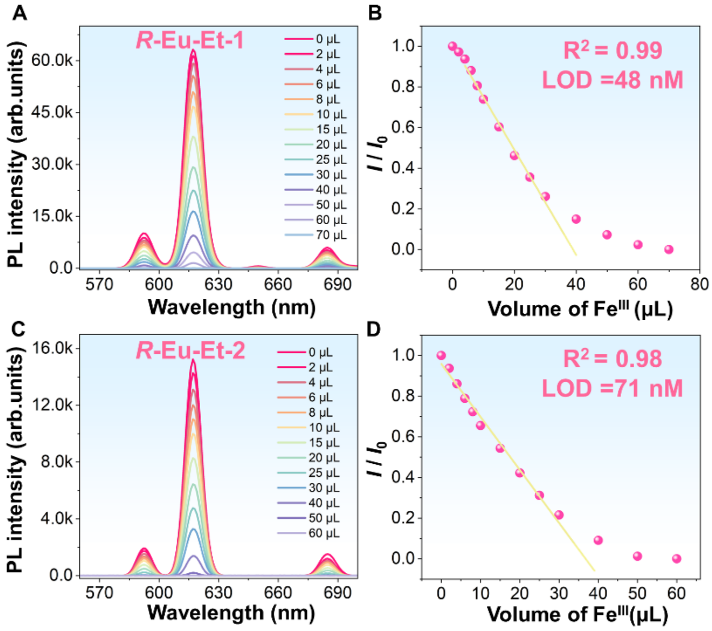

Supplementary Figure 37. **Sensing performance characterization**. Concentration-dependent spectra and fitting curves of ***R*-Eu-Et-1** (A and B) and ***R*-Eu-Et-2** (C and D) on Fe^{III} ions.

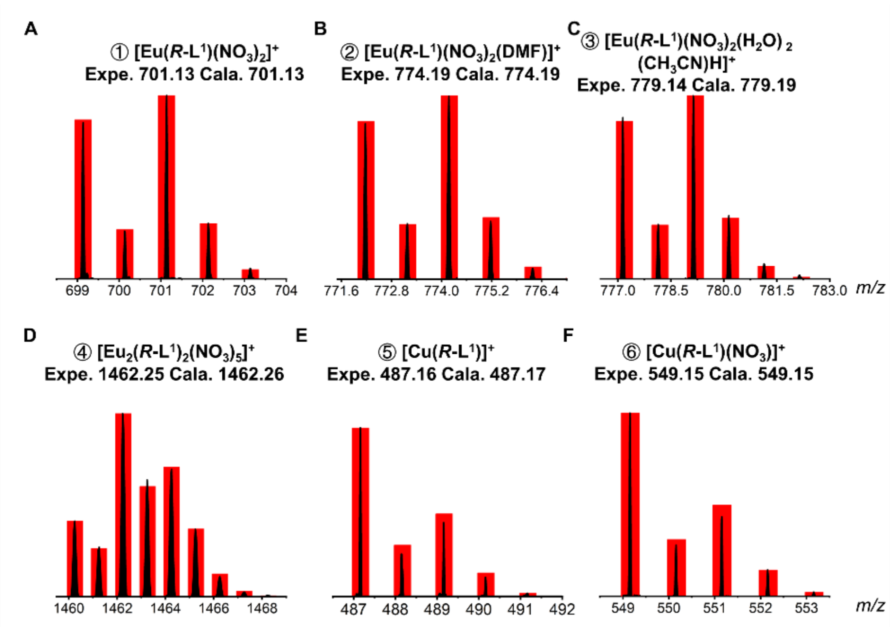

Supplementary Figure 38. **HRESI-MS characterization**. The fitting comparison of experimental and theoretical values of mass spectral molecular ion peaks of ***R*-Eu-Et-1** (A-D) and ***R*-Eu-Et-1** containing copper ions (E and F).

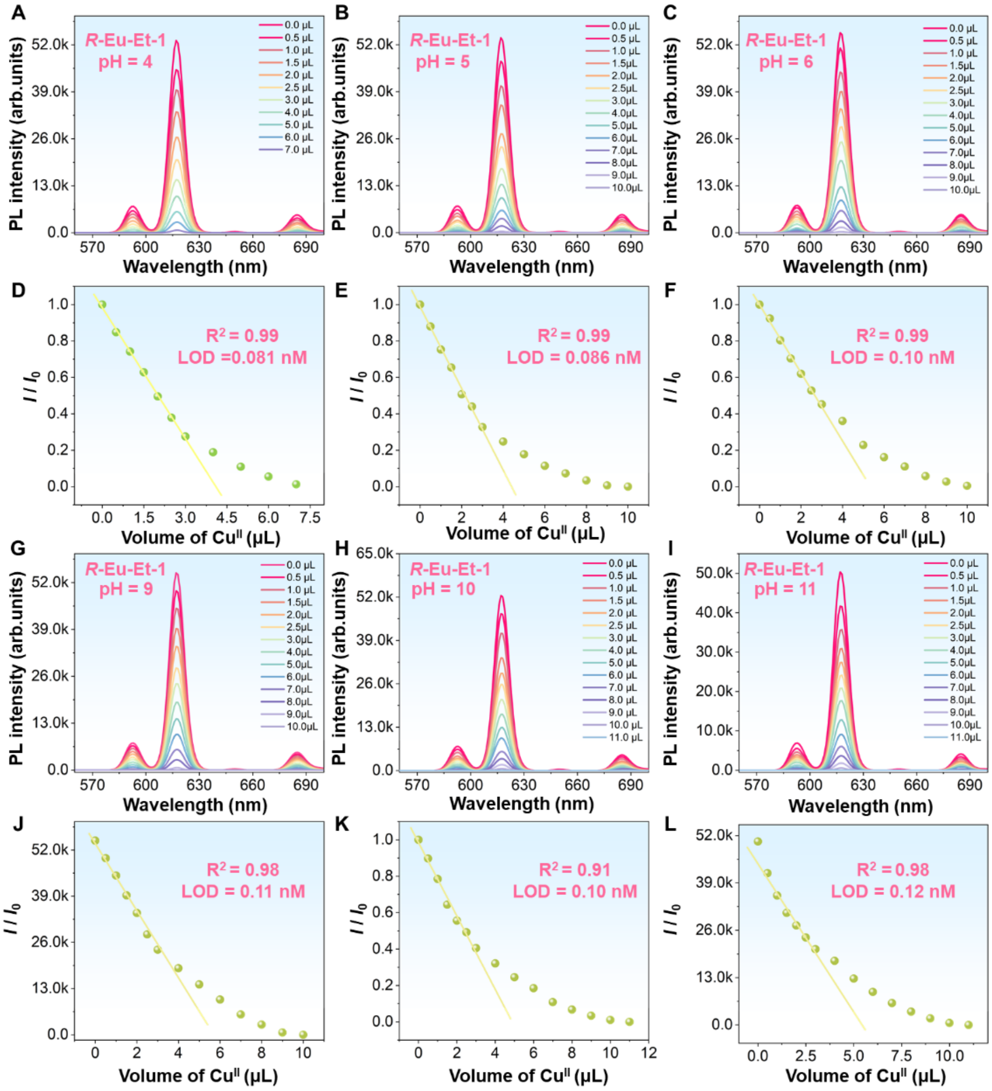

Supplementary Figure 39. **Sensing performance characterization**. Concentration-dependent spectra and fitting curves of ***R*-Eu-Et-1** on Cu^{II} ions under different pH conditions.

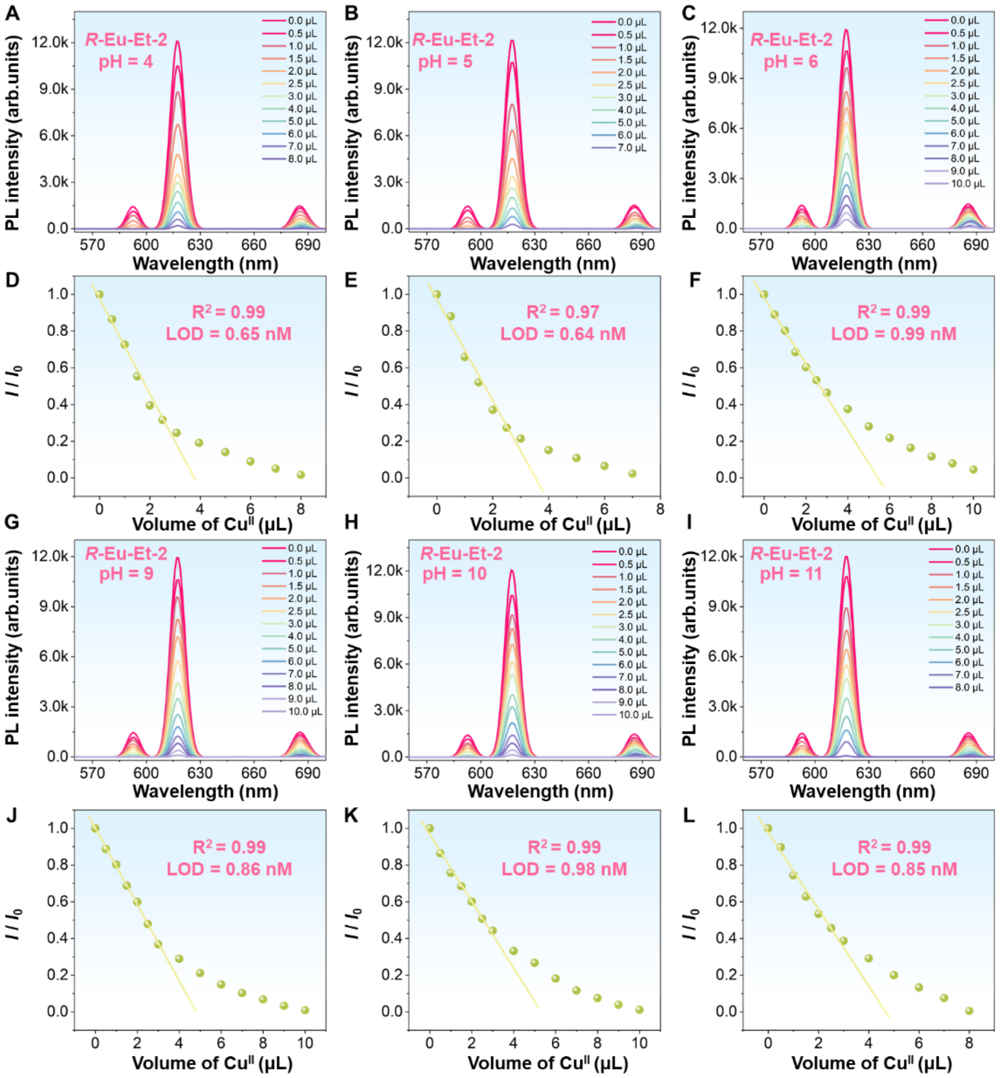

Supplementary Figure 40. **Sensing performance characterization**. Concentration-dependent spectra and fitting curves of ***R*-Eu-Et-2** on Cu^{II} ions under different pH conditions.

Supplementary Table 2. *SHAPE* analysis of the Eu^{III} in the ***R*/*S*-Eu-R-1** (R = Et/Me).

Label	Shape	Symmetry	Distortion (°)			
			Eu^{III}	Eu^{III}	Eu^{III}	Eu^{III}
DP-10	Decagon	D_{10h}	33.93532	34.01265	34.85338	34.80076
EPY-10	Enneagonal pyramid	C_{9v}	22.96372	23.03167	23.77974	23.73678
OBPY-10	Octagonal bipyramid	D_{8h}	16.11983	16.05873	15.91788	15.95144
PPR-10	Pentagonal prism	D_{5h}	11.88137	11.91474	11.90462	11.92960
PAPR-10	Pentagonal antiprism	D_{5d}	11.58262	11.65080	11.65807	11.67138
JBCCU-10	Bicapped cube	D_{4h}	9.10747	9.12625	8.88612	8.88208
JBCSAPR-10	Bicapped square antiprism	D_{4d}	3.38290	3.34359	3.46979	3.48286
JMBIC-10	Metabidiminished icosahedron	C_{2v}	7.48482	7.51418	7.61859	7.56531
JATDI-10	Augmented tridiminished icosahedron	C_{3v}	20.00186	20.01637	20.14091	20.15957
JSPC-10	Sphenocorona	C_{2v}	3.25670	3.27164	3.78984	3.78152

SDD-10	Staggered Dodecahedron	D_2	5.60124	5.64844	5.20931	5.23649
TD-10	Tetradecahedron	C_{2v}	4.81059	4.83076	4.22894	4.24507
HD-10	Hexadecahedron	D_{4h}	6.80745	6.84099	6.41746	6.38971

Supplementary Table 3. *SHAPE* analysis of the Eu^{III} in the ***R*/*S*-Eu-Et-2**.

Label	Shape	Symmetry	Distortion (°)	
			Eu^{III}	Eu^{III}
DP-10	Decagon	D_{10h}	34.55861	34.62324
EPY-10	Enneagonal pyramid	C_{9v}	23.75218	23.63423
OBPY-10	Octagonal bipyramid	D_{8h}	16.00718	15.97695
PPR-10	Pentagonal prism	D_{5h}	11.67462	11.70056
PAPR-10	Pentagonal antiprism	D_{5d}	11.15900	11.17049
JBCCU-10	Bicapped cube	D_{4h}	9.03325	9.07330
JBCSAPR-10	Bicapped square antiprism	D_{4d}	3.83080	3.86309
JMBIC-10	Metabidiminished icosahedron	C_{2v}	7.24120	7.22875
JATDI-10	Augmented tridiminished icosahedron	C_{3v}	19.29539	19.35434
JSPC-10	Sphenocorona	C_{2v}	3.35696	3.42123
SDD-10	Staggered Dodecahedron	D_2	4.80076	4.87477
TD-10	Tetradecahedron	C_{2v}	3.97508	4.03480
HD-10	Hexadecahedron	D_{4h}	6.43082	6.46030

Supplementary Table 4. *SHAPE* analysis of the Eu^{III} in the **Eu-Et-3**.

Label	Shape	Symmetry	Distortion (°)
			Eu^{III}
DP-10	Decagon	D_{10h}	35.40768
EPY-10	Enneagonal pyramid	C_{9v}	24.14090
OBPY-10	Octagonal bipyramid	D_{8h}	15.47770
PPR-10	Pentagonal prism	D_{5h}	12.15191
PAPR-10	Pentagonal antiprism	D_{5d}	10.74614
JBCCU-10	Bicapped cube	D_{4h}	8.39190
JBCSAPR-10	Bicapped square antiprism	D_{4d}	3.87471
JMBIC-10	Metabidiminished icosahedron	C_{2v}	6.90348
JATDI-10	Augmented tridiminished icosahedron	C_{3v}	19.26150
JSPC-10	Sphenocorona	C_{2v}	3.89992
SDD-10	Staggered Dodecahedron	D_2	4.77515
TD-10	Tetradecahedron	C_{2v}	3.73920
HD-10	Hexadecahedron	D_{4h}	5.63828

Supplementary Table 5. The fitting comparison of experimental and theoretical values of molecular ion peaks of *R*/*S*-L^1 and *R*/*S*-L^2.

	Peaks	Obs. *m./z*	Calc. *m/z*
I	$[(L^1)H^+]$	425.24	425.25
II	$[(L^1)(H_2O)H^+]$	443.26	443.25
III	$[(L^1)(H_2O)_2H^+]$	461.29	461.27
IV	$[(L^1)(H_2O)(CH_3OH)H^+]$	475.29	475.28
V	$[(L^1)(H_2O)_2(CH_3OH)H^+]$	439.31	493.29
VI	$[(L^2)H^+]$	327.23	327.23
VII	$[(L^2)Na^+]$	349.21	349.21

VIII	$[(L^2)(H_2O)(CH_3OH)_2H^+]$	409.31	409.29
IX	$[(L^2)(H_2O)_4(CH_3CN)]^+$	439.28	439.29
X	$[(L^2)(H_2O)_3(CH_3CN)(DMF)_3H^+]$	641.44	641.45

Supplementary Table 6. Tabulated photophysical data for ***R*/*S*-Eu-R-1** (**R** = **Et/Me**) and ***R*/*S*-Eu-Et-2**.

Compound	ε_λ	Radiative Lifetime (μs)	QY	Transition	$\|g_{lum}\|$	CPL Brightness B_{CPL} ($M^{-1}cm^{-1}$)
***R*-Eu-Et-1**	29190	1198	0.26	$^5D_0 \rightarrow ^7F_1$	0.64	2429
				$\rightarrow ^7F_2$	0.06	228
				$\rightarrow ^7F_3$	0.17	645
				$\rightarrow ^7F_4$	0.17	645
***S*-Eu-Et-1**	29190	1234	0.26	$^5D_0 \rightarrow ^7F_1$	0.62	2353
				$\rightarrow ^7F_2$	0.08	304
				$\rightarrow ^7F_3$	0.05	190
				$\rightarrow ^7F_4$	0.18	683
***R*-Eu-Me-1**	26426	1231	0.29	$^5D_0 \rightarrow ^7F_1$	0.62	2376
				$\rightarrow ^7F_2$	0.08	307
				$\rightarrow ^7F_3$	0.14	536
				$\rightarrow ^7F_4$	0.18	690
***S*-Eu-Me-1**	26426	1225	0.28	$^5D_0 \rightarrow ^7F_1$	0.49	1813
				$\rightarrow ^7F_2$	0.14	518
				$\rightarrow ^7F_3$	0.16	592
				$\rightarrow ^7F_4$	0.13	481
***R*-Eu-Et-2**	23636	1202	0.17	$^5D_0 \rightarrow ^7F_1$	0.06	121
				$\rightarrow ^7F_2$	0.02	40
				$\rightarrow ^7F_3$	0.16	321
				$\rightarrow ^7F_4$	0.05	100
***S*-Eu-Et-2**	22121	1241	0.17	$^5D_0 \rightarrow ^7F_1$	0.08	150
				$\rightarrow ^7F_2$	0.02	38
				$\rightarrow ^7F_3$	0.17	320
				$\rightarrow ^7F_4$	0.05	94

Supplementary Table 7. B_{CPL} value of chiral Eu^{III} complexes.

Eu^{III} complexes	B_{CPL} (M^{-1} cm^{-1})	Ref.
$Eu_4L_4(L')_4$ Cage	3240 and 1122	2
$EuL^7(HFA)_3$	0.7 and 1	3
$Eu(L^8)_3$	1.3 and 1.7	4
$EuL^9(HFA)_3$	3.4 and 1.7	3
$Eu(HL^6)(H_2O)$	9.7	5
$EuL^{10}(L'')$	14.3	6
$[EuL^{11}]^{3+}$	29.6 and 46.4	7
$[EuL^{12}]^{3+}$	35.1 and 50	7
$[Eu(H_2L^4)]^+$	45.5	8
$CsEu(hfbc)_4$	50.7 and 59.1	9 and 10
$EuL^{14}(TTA)_3$	86.6 and 64.7	11
$[Eu_3(L^5)_6]^{2+}$	100.7 and 171.9	12
$[EuL_3]\cdot 3Cl$	102 and 213	13
$EuL^{16}(TTA)_3$	103.7 and 84.2	11
EuL^{17}	116	14
EuL^{19}	134.5	14
$[Eu\cdot L^4]$	117	14
EuL^{20}	133.1	15

This work	2429	

Supplementary Data 1. Selected bond lengths (Å) and angles (°) of ***R***/***S*-Eu-R-1** (R = Et/Me), ***R***/***S*-Eu-Et-2**, **Eu-Et-3**, ***R***/***S*-Gd-R-1** (R = Et/Me), ***R*-Gd-Et-2**, ***R*-Tb-Et-1**, ***R***/***S*-Tb-Me-1**, and ***R*-Tb-Et-2**.

Supplementary Data 2. QYs of ***R***/***S*-Eu-Et-1**, ***R***/***S*-Eu-Et-1**, and ***R***/***S*-Eu-Et-2** dispersed in glycerin/DMSO or CH_3CN/DMF mixtures with different f_w.

Supplementary Data 3. DFT-computed coordinates of ***R*-Eu-Et-1**.

Supplementary Data 4. DFT-computed coordinates of ***R*-Eu-Et-2**.

Supplementary Reference

1. Sheldrick, G. M. Crystal structure refinement with SHELXL. *Acta Crystallogr., Sect. C: Struct. Chem*. **71**, 3–8 (2015).
2. Zhou, Y., Li, H., Zhu, T., Gao, T. & Yan, P. A highly luminescent chiral tetrahedral $Eu_4L_4(L')_4$ cage: Chirality induction, chirality memory, and circularly polarized luminescence. *J. Am. Chem. Soc.* **141**, 19634–19643 (2019).
3. Arrico, L., Di Bari, L. & Zinna, F. Quantifying the Overall Efficiency of Circularly Polarized Emitters. *Chem. Eur. J.* **27**, 2920–2934 (2021).
4. Article, E. *et al.* The application of chiroptical spectroscopy (circular dichroism) in quantifying binding events in lanthanide directed synthesis of chiral luminescent self-assembly structures. *Chem. Sci.*, **6**, 457–471 (2015).
5. Seitz, M., Moore, E. G., Ingram, A. J., Muller, G. & Raymond, K. N. Enantiopure, octadentate ligands as sensitizers for europium and terbium circularly polarized luminescence in aqueous solution. *J. Am. Chem. Soc.* **129**, 15468–15470 (2007).
6. Liu, D. *et al.* Chiral BINAPO-Controlled Diastereoselective Self-Assembly and Circularly Polarized Luminescence in Triple-Stranded Europium(III) Podates. *Inorg. Chem.* **57**, 8332–8337 (2018).
7. Hasegawa, M. *et al.* Chiroptical Spectroscopic Studies on Lanthanide Complexes with Valinamide Derivatives in Solution. *Chempluschem* **85**, 294–300 (2020).
8. Lama, M. *et al.* Lanthanide class of a trinuclear enantiopure helical architecture containing chiral ligands: Synthesis, structure, and properties. *Chem. Eur. J.* **13**, 7358–7373 (2007).
9. Zinna, F., Giovanella, U. & Bari, L. Di. Highly circularly polarized electroluminescence from a chiral europium complex. *Adv. Mater.* **27**, 1791–1795 (2015).
10. Taniguchi, A. *et al.* Circularly polarised luminescence from planar-chiral Phanephos/Tb(III)$(hfa)_3$ hybrid luminophores. *Photochem. Photobiol. Sci.* **18**, 2859–2864 (2019).
11. Górecki, M., Carpita, L., Arrico, L., Zinna, F. & Di Bari, L. Chiroptical methods in a wide

wavelength range for obtaining Ln^{3+} complexes with circularly polarized luminescence of practical interest. *Dalton Trans.* **47**, 7166–7177 (2018).

12. Petoud, S. *et al.* Brilliant Sm, Eu, Tb, and Dy chiral lanthanide complexes with strong circularly polarized luminescence. *J. Am. Chem. Soc.* **129**, 77–83 (2007).
13. Starck, M., Mackenzie, L. E., Batsanov, A. S., Parker, D. & Pal, R. Excitation modulation of Eu:BPEPC based complexes as low-energy reference standards for circularly polarised luminescence (CPL). *Chem. Commun.* **55**, 14115–14118 (2019).
14. Frawley, A. T., Pal, R. & Parker, D. Very bright, enantiopure Europium(III) complexes allow time-gated chiral contrast imaging. *Chem. Commun.* **52**, 13349–13352 (2016).
15. Butler, S. J. *et al.* Utility of tris(4-bromopyridyl) europium complexes as versatile intermediates in the divergent synthesis of emissive chiral probes. *Dalton Trans.* **43**, 5721–5730 (2014).
