# Supplementary material for: Aggregation induced emission dynamic chiral europium(III) complexes with excellent circularly polarized luminescence and smart sensors
Source: Nat Commun. 2024 Apr 4;15:2896. doi: 10.1038/s41467-024-47246-z (PMC10994944; doi:10.1038/s41467-024-47246-z)
Supplement: Supplementary file 3 — Description of Additional Supplementary Files [file 41467_2024_47246_MOESM3_ESM.pdf]

Description of Additional Supplementary Files

File Name: Supplementary Data 1
Description: Selected bond lengths (Å) and angles (°) of ***R/S*-Eu-R-1** (R = Et/Me), ***R/S*-EuEt-2**, **Eu-Et-3**, ***R/S*-Gd-R-1** (R = Et/Me), ***R*-Gd-Et-2**, ***R*-Tb-Et-1**, ***R/S*-Tb-Me-1**, and ***R*-Tb-Et-2**.

File Name: Supplementary Data 2
Description: QYs of ***R/S*-Eu-Et-1**, ***R/S*-Eu-Et-1**, and ***R/S*-Eu-Et-2** dispersed in glycerin/DMSO or CH3CN/DMF mixtures with different fw.

File Name: Supplementary Data 3
Description: DFT-computed coordinates of ***R*-Eu-Et-1.**

File Name: Supplementary Data 4
Description: DFT-computed coordinates of ***R*-Eu-Et-2.**

File Name: Supplementary Movie 1
Description: *R*-Eu-Et-1-original video.

File Name: Supplementary Movie 2
Description: *R*-Eu-Et-2-original video.

File Name: Supplementary Movie 3
Description: *R*-Eu-Et-1-slowed down 10 times.

File Name: Supplementary Movie 4
Description: *R*-Eu-Et-2-slowed down 10 times.
